# Acute Effects of Smoked and Vaporized Cannabis in Healthy Adults Who Infrequently Use Cannabis: A Crossover Trial

**DOI:** 10.1001/jamanetworkopen.2018.4841

**Published:** 2018-11-30

**Authors:** Tory R. Spindle, Edward J. Cone, Nicolas J. Schlienz, John M. Mitchell, George E. Bigelow, Ronald Flegel, Eugene Hayes, Ryan Vandrey

**Affiliations:** 1 Behavioral Pharmacology Research Unit, Johns Hopkins University School of Medicine, Baltimore, Maryland; 2 RTI International, Research Triangle Park, North Carolina; 3 Division of Workplace Programs, Substance Abuse and Mental Health Services Administration, Rockville, Maryland

## Abstract

**Importance:**

Vaporization is an increasingly popular method for cannabis administration, and policy changes have increased adult access to cannabis drastically. Controlled examinations of cannabis vaporization among adults with infrequent current cannabis use patterns (>30 days since last use) are needed.

**Objective:**

To evaluate the acute dose effects of smoked and vaporized cannabis using controlled administration methods.

**Design, Setting, and Participants:**

This within-participant, double-blind, crossover study was conducted from June 2016 to January 2017 at the Behavioral Pharmacology Research Unit, Johns Hopkins University School of Medicine, and included 17 healthy adults. Six smoked and vaporized outpatient experimental sessions (1-week washout between sessions) were completed in clusters (order counterbalanced across participants); dose order was randomized within each cluster.

**Interventions:**

Cannabis containing Δ9-tetrahydrocannabinol (THC) doses of 0 mg, 10 mg, and 25 mg was vaporized and smoked by each participant.

**Main Outcomes and Measures:**

Change from baseline scores for subjective drug effects, cognitive and psychomotor performance, vital signs, and blood THC concentration.

**Results:**

The sample included 17 healthy adults (mean [SD] age, 27.3 [5.7] years; 9 men and 8 women) with no cannabis use in the prior month (mean [SD] days since last cannabis use, 398 [437] days). Inhalation of cannabis containing 10 mg of THC produced discriminative drug effects (mean [SD] ratings on a 100-point visual analog scale, smoked: 46 [26]; vaporized: 69 [26]) and modest impairment of cognitive functioning. The 25-mg dose produced significant drug effects (mean [SD] ratings, smoked: 66 [29]; vaporized: 78 [24]), increased incidence of adverse effects, and pronounced impairment of cognitive and psychomotor ability (eg, significant decreased task performance compared with placebo in vaporized conditions). Vaporized cannabis resulted in qualitatively stronger drug effects for most pharmacodynamic outcomes and higher peak concentrations of THC in blood, compared with equal doses of smoked cannabis (25-mg dose: smoked, 10.2 ng/mL; vaporized, 14.4 ng/mL). Blood THC concentrations and heart rate peaked within 30 minutes after cannabis administration and returned to baseline within 3 to 4 hours. Several subjective drug effects and observed cognitive and psychomotor impairments persisted for up to 6 hours on average.

**Conclusions and Relevance:**

Vaporized and smoked cannabis produced dose-orderly drug effects, which were stronger when vaporized. These data can inform regulatory and clinical decisions surrounding the use of cannabis among adults with little or no prior cannabis exposure.

**Trial Registration:**

ClinicalTrials.gov Identifier: NCT03676166.

## Key Points

**Question:**

How does smoked and vaporized cannabis acutely influence subjective drug effects, cognitive and psychomotor performance, and cardiovascular measures in healthy adults who infrequently use cannabis (>30 days since last use)?

**Findings:**

In a crossover trial of 17 healthy adults, inhalation of smoked and vaporized cannabis containing 10 mg of Δ9-tetrahydrocannabinol (THC) produced discriminative drug effects and modest impairment of cognitive functioning, while inhalation of a 25-mg dose of THC was associated with pronounced drug effects, increased incidence of adverse effects, and significant impairment of cognitive and psychomotor ability. Vaporized cannabis produced greater pharmacodynamic effects and higher concentrations of THC in blood compared with equal doses of smoked cannabis.

**Meaning:**

Significant, sometimes adverse, drug effects can occur at relatively low THC doses in infrequent cannabis users, and accordingly these data should be considered with regard to regulation of retail cannabis products and education for individuals initiating cannabis use.

## Teaser

This crossover study evaluates the acute effects of smoked vs vaporized cannabis use, at multiple Δ9-tetrahydrocannabinol (THC) doses, among healthy adults who infrequently use cannabis.

## Introduction

Cannabis (marijuana) policy and regulation are under dramatic reform throughout the developed world. At the time of this writing, medicinal use of cannabis was approved in 30 US states and Washington, DC, and nonmedicinal use was permitted in 9 states. Numerous countries in the European Union and elsewhere have also approved cannabis for medicinal (eg, Australia) and nonmedicinal (eg, Uruguay and Canada^1^) use. Corresponding with these policy changes, perceived harm associated with cannabis use has decreased.^2,3^ These changes have also spawned a new retail cannabis marketplace, which has increased access to cannabis and driven the development of numerous novel cannabis products and formulations.

Historically, cannabis has predominantly been smoked using various implements such as joints, pipes, bongs, and blunts.^4^ Assorted vaporizers, analogous to electronic cigarettes, have emerged^5^ and become an increasingly popular method for cannabis administration,^6,7^ particularly in states permitting nonmedicinal use of cannabis (eg, California^8^). Cannabis vaporizers heat dried cannabis or concentrated cannabis extracts and/or resins, creating an inhalable aerosol or vapor.^9^ Vaporization is associated with less toxicant exposure (eg, polycyclic aromatic hydrocarbons) relative to traditional smoking methods,^10,11^ which increases product appeal.^6,7^

In most prior controlled laboratory studies of acute cannabis effects, daily or near daily cannabis users have self-administered smoked cannabis. Consequently, the comparative acute effects of smoked vs vaporized cannabis and individual responses to acute cannabis exposure have not been sufficiently characterized for infrequent cannabis users. The few studies directly comparing the acute effects of smoked and vaporized cannabis have generally revealed similar pharmacokinetic (eg, blood Δ9-tetrahydrocannabinol [THC] concentrations) and pharmacodynamic (eg, subjective ratings of “high”) profiles across these 2 methods.^12,13,14^ However, limitations of extant studies have included the use of single THC doses, relatively low THC concentrations in the plant material (1.7%-6.9% THC), small sample sizes, and/or use of uniform puffing procedures (ie, 5-second inhalations followed by a 10-second breath hold) that may be inconsistent with naturalistic puffing profiles and do not fully account for individual differences in puff topography that can produce variation in dose delivery. Furthermore, the extent to which cognitive and psychomotor impairment differs as a function of cannabis inhalation method (ie, smoked vs vaporized) has not been systematically evaluated. Given the increased popularity of vaporization and increased access to cannabis in the expanding medicinal and nonmedicinal markets, controlled studies comparing the acute effects of smoked and vaporized cannabis administration among infrequent cannabis users are vital, and may inform dosing guidelines, cannabis policy and regulation, and procedures for detecting acute cannabis intoxication.

The goal of this study was to compare the pharmacodynamics and pharmacokinetics of smoked and vaporized cannabis in healthy adults. This study extends prior research by examining multiple doses of THC across inhalation methods, enrolling individuals with infrequent cannabis use patterns (defined here as no use in the past 30 days accompanied with a negative urine toxicology test result), and including a comprehensive pharmacodynamic test battery (ie, subjective drug effects, cognitive and psychomotor performance, and vital signs).

## Methods

### Participants

Study volunteers were recruited via advertisements and word of mouth. Eligible participants were deemed healthy by medical history review, electrocardiogram, blood testing (hematology and serology), and a physical examination. Participants self-reported prior use of cannabis but denied use of cannabis or other illicit drugs in the month prior to participation (assessed with timeline follow-back method^15^). Urine toxicology testing for cannabis, amphetamines, benzodiazepines, cocaine, 3,4-methylenedioxymethamphetamine (MDMA), opioids, and phencyclidine was performed using rapid enzyme immunoassay test kits at screening and prior to each experimental session; participants were required to test negative for all drugs, including cannabis, before each session. This study was approved by the Johns Hopkins Medicine institutional review board and all participants provided written informed consent.

### Study Design and Procedure

**Figure 1.**
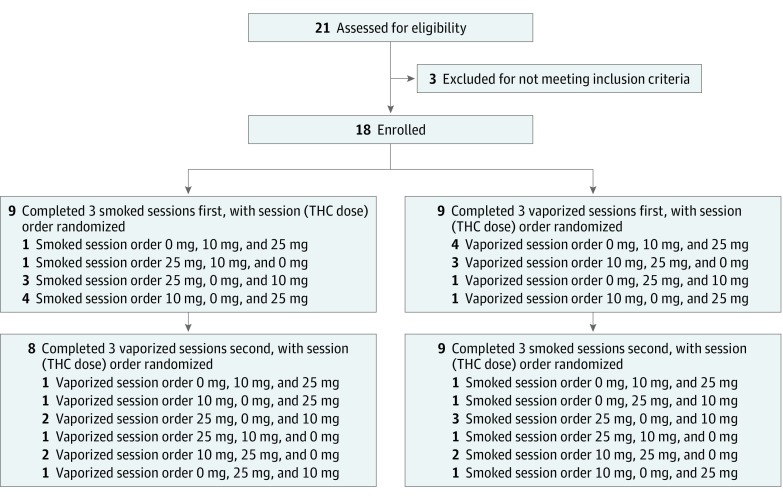
CONSORT Flow Diagram THC indicates Δ9-tetrahydrocannabinol.

This within-individuals, double-blind, crossover study was conducted at the Johns Hopkins Behavioral Pharmacology Research Unit and followed the Consolidated Standards of Reporting Trials (CONSORT) reporting guideline (Trial Protocol in Supplement 1). A double-dummy procedure to blind participants and research staff to inhalation method was not used in order to equally capture peak drug effects that occur immediately after inhalation. All participants completed six 8.5-hour outpatient sessions that differed only by inhalation method (smoked vs vaporized) and THC dose (0 mg, 10 mg, or 25 mg). All participants were compensated for their time. Sessions were separated by at least 1 week and clustered by inhalation method (ie, cannabis was smoked for the first 3 sessions and vaporized the final 3 sessions or vice versa). The order of inhalation method was counterbalanced across participants (ie, half of participants completed smoked sessions first and the other half completed vaporized sessions first). The THC dose order was randomized within each inhalation method cluster (Figure 1).

At the start of each session, participants completed a urine drug screening and alcohol breathalyzer to confirm compliance with instructions to not use illicit drugs or alcohol; female participants also completed a urine pregnancy test. An intravenous catheter was placed in a forearm vein of the nondominant arm and a baseline blood sample was collected. Additional baseline assessments of heart rate (HR), blood pressure (BP), cognitive and psychomotor performance, and subjective drug effects were obtained. Following baseline assessments, participants self-administered the assigned cannabis dose by inhaling the study product ad libitum within a 10-minute period. During vaporized cannabis sessions, the Volcano Medic (Storz & Bickel, Oakland, California) was used to heat and aerosolize the cannabis, which was then trapped in a balloon and given to participants to inhale ad libitum until the balloon was empty. To ensure complete vaporization of the highest dose, participants inhaled 3 balloons within the designated 10-minute period. A new balloon was used for each experimental session to avoid contamination from prior doses. During smoked cannabis sessions, participants were given a small handheld pipe prefilled with cannabis and given 10 minutes to self-administer the entire dose by igniting the plant material with a lighter and inhaling the resulting smoke. To more effectively blind participants and study staff, an opaque bag was used to cover the vaporizer balloons and thus decrease the visibility of the vapor inside, and the pipe was fitted with a metal top to conceal the plant material; the metal top also minimized drug loss owing to sidestream smoke. Unblinded research pharmacy staff visually inspected the contents of the pipe to ensure complete dose consumption.

### Study Drug

Cannabis used in this study was obtained from the National Institute on Drug Abuse Drug Supply Program. Cannabis was weighed before each session to deliver target THC doses of 0 mg, 10 mg, and 25 mg. Two batches of cannabis were used. Batch 1 contained 13.4% Δ9-THC, 0.08% Δ8-THC, 0.03% cannabidiol (CBD), and 0.8% cannabinol. Batch 2 (placebo) contained less than 0.01% Δ9-THC and had no detectable concentrations of Δ8-THC, CBD, or cannabinol. The same amount of plant material was placed into the pipe or vaporizer for each session. For the 0-mg condition, 186.6 mg of batch 2 cannabis was used; for the 10-mg condition, 74.6 mg of batch 1 and 112 mg of batch 2 cannabis were mixed together; and for the 25-mg condition, 186.6 mg of batch 1 cannabis was used.

### Outcome Measures

A battery of assessments was administered at baseline and at 0.17, 0.5, 1, 1.5, 2, 3, 4, 5, 6, and 8 hours after drug administration during each session. Blood was sampled and HR and BP were measured at the same time points. Cognitive performance tasks were omitted at the 0.17-hour time point owing to time limitations.

#### Subjective Drug Effects

Subjective drug effects were assessed with the Drug Effect Questionnaire (DEQ).^16^ The DEQ uses a 100-mm visual analog scale with the horizontal line anchored with 0 (or not at all) on the left and 100 (or extremely) on the right. Items assessed the extent to which participants felt the following: drug effects, pleasant drug effects, unpleasant drug effects, sick, heart racing, anxious and/or nervous, relaxed, paranoid, alert, irritable, vigorous and/or motivated, restless, hungry and/or had the munchies, sleepy, dry mouth, dry, red, and/or irritated eyes, throat irritation and/or coughing, difficulty performing routine tasks, memory impairment, and cravings from cannabis.

#### Cognitive, Psychomotor, and Cardiovascular Measures

Cognitive and psychomotor performance was assessed using 3 computerized tasks previously demonstrated to be acutely influenced by cannabis self-administration and representative of workplace performance and/or operation of a motor vehicle.^17,18,19^ These tasks included the following: (1) the Digit Symbol Substitution Task (DSST^20^) in which participants replicated the shape of patterns presented on their screen using a computer keyboard (primary outcomes: number of patterns attempted, number correct, and accuracy within the 90 allocated seconds), (2) the Divided Attention Task (DAT^21^) where participants performed a central motor task (tracking a stimulus with a mouse cursor moving horizontally on a screen at a fixed speed) while simultaneously responding to peripherally located stimuli on the screen (primary outcomes: mean distance of the cursor from the central target stimulus, number of peripheral stimuli identified correctly out of 24 administered, and response time for recognition of peripheral stimuli), and (3) a computerized version of the Paced Auditory Serial Addition Task (PASAT^22^) where participants viewed a string of single-digit numbers and attempted to select the sum of the 2 numbers most recently presented on the screen (primary outcomes: total number of correct trials out of 90 administered; reaction time for correct and incorrect responses). Unless specified, the range of scores for these outcomes was not fixed. Participants received training on these tasks during the screening evaluation to establish a baseline and lower practice effects during the sessions. The participants’ HR and systolic and diastolic BP were measured in the seated position using an automated monitor.

#### Blood Specimens

Blood samples were collected using 10-mL gray-top vacutainer tubes. Whole-blood concentration of THC was measured by Immunalysis Corporation using liquid chromatography–tandem mass spectrometry (LC-MS/MS), limit of quantitation (LOQ) of 0.5 ng/mL, and upper limit of linearity (ULOL) of 100 ng/mL.^18,23^

### Statistical Analysis

A meta-analysis^24^ conducted on 6 acute drug administration studies (each with 14 participants and a range of drug doses) determined that average effect sizes for primary outcome measures (eg, subjective drug effects and cognitive assessments) ranged from 0.87 to 1.0, indicating that the sample size for the current study (N = 17) was sufficient. Demographic characteristics and whole-blood THC data were presented using descriptive statistics including means and standard deviations. Data for vital signs, subjective drug effects, and cognitive and psychomotor performance were analyzed using repeated-measures regressions (covariance structure: first order autoregressive). Separate regressions were conducted on each outcome with 3 factors included in each model: time (change from baseline scores), dose (0 mg, 10 mg, and 25 mg), and inhalation method (smoked vs vaporized). Planned contrasts between placebo (0 mg) and active doses (10 mg and 25 mg) within each inhalation method (smoked and vaporized) and between inhalation methods at each active dose were conducted using peak change from baseline scores for each variable. Correlations were conducted to examine the relation between change from baseline scores for blood THC concentrations and change from baseline scores for the item drug effect (from the DEQ), HR, and primary DSST, DAT, and PASAT outcomes. For all analyses, statistical significance was defined as an α error probability level of less than .05. Several steps were taken to lower familywise error rate. For correlations, α levels were adjusted using the Holm-Bonferroni method.^25^ For each nonorthogonal set of planned contrasts, (ie, those that compared 0 mg with both 10 mg and 25 mg), a Bonferroni correction was applied. Because 2 comparisons were made to the 0-mg condition within each inhalation method for each outcome measure, the threshold for statistical significance for these planned contrasts was set to a *P* value less than .025. Since the other series of planned contrasts between smoked and vaporized conditions at each dose were orthogonal in nature, no α corrections were applied.^26^ Analyses were conducted in SAS, version 9.4 (PROC MIX; SAS Institute) and SPSS statistical software, version 23 (IBM Inc).

## Results

Seventeen healthy adult participants (9 men and 8 women) completed the study. The mean (SD) age of these individuals was 27.3 (5.7) years and their mean (SD) weight and body mass index (calculated as weight in kilograms divided by height in meters squared) were 77.9 (15.5) kg and 26.2 (3.3), respectively. Self-reported races and ethnicities for study completers were as follows: 10 white or non-Hispanic, 3 other or Hispanic, 3 black or non-Hispanic, and 1 white or Hispanic. A mean (SD) of 398 (437) days had passed (median [range] days, 365 [30-1825] days) since last self-reported cannabis use at the time of study entry.

### Subjective Drug Effects

**Figure 2.**
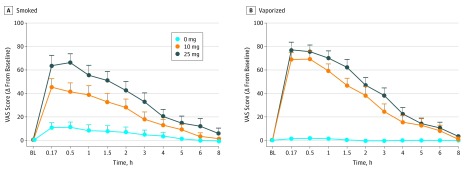
Mean Ratings for Visual Analog Scale (VAS) Item Drug Effect From the Drug Effect Questionnaire Displayed Over Time and Across Δ9-Tetrahydrocannabinol Dose for Smoked and Vaporized Conditions Scores ranged from 0 (not at all) to 100 (extremely). Error bars indicate SEM. Horizontal axes are not accurate time scales and represent the time points at which subjective drug effects were assessed. BL indicates baseline time point; and Δ, difference or change.

For both smoked and vaporized cannabis inhalation, numerous drug effects were significantly greater in the active-dose conditions (ie, 10 mg and 25 mg of THC) compared with placebo (mean peak change from baseline scores, time of peak change from baseline, and indicators of statistical significance) (Table). For both inhalation methods, mean peak changes for ratings of drug effect at the 10-mg and 25-mg doses were significantly greater than placebo (*P* < .025; Figure 2); the same trend was observed for pleasant, sleepy, hungry or had the munchies, and dry mouth (all *P* values <.025). At the 10-mg and 25-mg dose for both inhalation methods, increased mean (SD) ratings of heart racing (vaporized: 10 mg, 16.4 [20.2]; 25 mg, 24.2 [29.1]; smoked: 10 mg, 4.2 [10.2]; 25 mg, 17.9 [23.7]) and difficulty performing routine tasks (vaporized: 10 mg, 26.0 [33.2]; 25 mg, 34.2 [30.6]; smoked: 10 mg, 12.8 [27.7]; 25 mg, 30.6 [36.3]) were observed relative to placebo (all *P* values <.025). At the 25-mg dose, vaporized and smoked cannabis increased mean (SD) ratings of unpleasant (vaporized: 24.4 [32.4]; smoked: 32.9 [34.8]), anxious and/or nervous (vaporized: 25.5 [28.0]; smoked: 21.4 [32.2]), memory impairment (vaporized: 16.2 [27.4]; smoked: 14.2 [27.1]), and throat irritation and/or coughing (vaporized: 22.18 [27.6]; smoked: 27.8 [25.5]) relative to placebo (all *P* values <.025). Mean (SD) ratings of dry and/or red eyes increased for the 10-mg (19.2 [28.9]) and 25-mg (25.1 [27.7]) vaporized cannabis doses compared with placebo (all* P* values <.025). The 25-mg dose of vaporized cannabis increased ratings of paranoid (mean [SD], 17.4 [30.0]) and the 25-mg dose of smoked cannabis increased ratings of sick (mean [SD], 20.3 [32.7])compared with placebo (all *P* values <.025).

**Table.** Mean Peak Change From Baseline Values for Pharmacodynamic Measures by Inhalation Method and THC Dose

Characteristic	Smoked						Vaporized					
	0-mg THC		10-mg THC		25-mg THC		0-mg THC		10-mg THC		25-mg THC	
	Peak Change, Mean (SD)	Peak Time, h^a^	Peak Change, Mean (SD)	Peak Time, h^a^	Peak Change, Mean (SD)	Peak Time, h^a^	Peak Change, Mean (SD)	Peak Time, h^a^	Peak Change, Mean (SD)	Peak Time, h^a^	Peak Change, Mean (SD)	Peak Time, h^a^
Subjective measures												
DEQ, mean (SD)^b^												
Drug effect	11.2 (15.9)	0.5	45.7 (26.4)^c^	0.17	66.4 (28.6)^c^	0.5	2.1 (5.2)	0.5	69.5 (26.4)^c^^,^^d^	0.5	77.5 (23.4)^c^	0.17
Unpleasant	2.8 (7.3)	0.17	12.8 (20.4)	0.17	32.9 (34.8)^c^	0.5	0.3 (1.2)	0.17	19.4 (24.7)	0.5	24.4 (32.4)^c^	1
Pleasant	10.2 (16.0)	0.17	42.4 (31.6)^c^	0.17	44.2 (31.2)^c^	0.17	1.2 (4.9)	1	59.2 (29.6)^c^	0.5	57.4 (26.8)^c^	0.17
Sick	2.2 (6.6)	0.17	2.3 (4.5)	0.17	20.3 (32.7)^c^	0.17	0.9 (3.6)	0.17	8.4 (33.0)	0.17	14.9 (20.5)	0.5
Heart racing	0.9 (3.1)	0.17	4.2 (10.2)	0.17	17.9 (23.7)^c^	0.17	−2.1 (6.0)	0.5	16.4 (20.2)^c^	0.17	24.2 (29.1)^c^	0.17
Anxious/nervous	−3.3 (8.2)	0.5	3.1 (10.5)	0.17	21.4 (32.2)^c^	0.17	−9.3 (15.2)	2	−3.6 (7.4)	5	25.5 (28.0)^c^	0.5
Relaxed	−4.1 (24.3)	8	23.5 (29.0)	1	9.8 (27.2)	1.5	9.0 (27.2)	0.17	13.8 (32.7)	1.5	−6.4 (30.3)	0.5
Paranoid	0.1 (0.5)	0.17	5.5 (17.7)	0.5	10.0 (22.0)	0.5	0.0 (0.0)	NA	7.9 (16.9)	0.17	17.4 (30.0)^c^^,^^d^	0.5
Sleepy	−21.6 (33.2)	8	25.1 (31.3)^c^	4	29.6 (29.2)^c^	2	−23.4 (27.1)	8	20.5 (39.2)^c^	1.5	31.3 (42.0)^c^	1
Alert	−13.1 (22.7)	1.5	−28.8 (35.5)^c^	1	−20.8 (31.7)	1.5	−11.5 (24.4)	4	−20.4 (28.3)	1.5	−26.0 (22.5)	2
Irritable	5.2 (22.1)	1.5	−2.8 (8.7)	1.5	10.1 (18.1)	1	1.6 (6.5)	4	3.3 (10.0)	1	7.7 (18.7)	0.5
Vigorous/motivated	−5.9 (16.3)	1.5	−8.2 (24.1)	2	−15.59 (33.1)	1.5	6.9 (18.0)	1	−21.8 (29.4)^c^^,^^d^	4	10.0 (29.7)^d^	8
Restless	8.1 (25.1)	8	5.8 (12.7)	8	14.1 (28.3)	0.5	9.1 (19.3)	8	10.5 (21.2)	0.17	13.6 (27.0)	0.5
Hungry/had munchies	13.6 (28.0)	3	23.6 (38.2)^c^	3	33.1 (31.4)^c^	3	12.2 (25.6)	3	34.2 (32.5)^c^	3	38.4 (30.3)^c^	3
Craving	0.0 (0.0)	NA	1.0 (4.1)	2	1.1 (2.9)	2	0.0 (0.0)	NA	3.4 (13.8)	1.5	−1.2 (5.1)	0.17
Dry mouth	5.7 (16.5)	6	38.5 (32.8)^c^	0.17	42.6 (32.8)^c^	0.5	2.5 (10.6)	0.17	61.8 (23.6)^c^^,^^d^	0.17	67.1 (27.8)^c^	0.5
Dry/red eyes	2.9 (10.6)	1.5	12.7 (23.1)	0.17	15.8 (20.8)	0.17	1.6 (6.8)	2	19.2 (28.9)^c^^,^^d^	1.5	25.1 (27.7)^c^	0.5
Memory impairment	2.1 (10.1)	0.5	6.5 (14.3)	0.5	14.2 (27.1)^c^	1.5	0.2 (0.9)	1.5	12.9 (18.0)	1	16.2 (27.4)^c^	1
Throat irritation/coughing	7.4 (10.2)	0.17	16.6 (31.2)	0.17	27.8 (25.5)^c^	0.17	1.2 (5.1)	2	9.0 (15.6)	0.17	22.18 (27.6)^c^	0.17
Difficulty performing routine tasks	3.2 (13.2)	1.5	12.8 (27.7)	1.5	30.6 (36.3)^c^	0.5	−0.4 (1.7)	2	26.0 (33.2)^c^	0.5	34.2 (30.6)^c^	0.5
Cognitive measures												
DSST, mean (SD)												
Total attempted, No.	−2.8 (11.3)	4	−1.6 (5.5)	1	−2.8 (6.4)	1	4.2 (6.4)	4	−6.0 (10.0)^c^	1	−10.0 (12.7)^c^^,^^d^	0.5
Total correct, No.	−3.2 (12.3)	4	3.4 (4.4)	8	−7.0 (14.3)	2	4.9 (8.9)	4	−8.3 (11.3)^c^	0.5	−13.8 (14.9)^c^^,^^d^	1
% Correct	−2 (6)	3	−4 (8)	2	−10 (24)	2	2 (13)	0.5	−7 (13)^c^	0.5	−16 (28)^c^	1
DAT												
Time tracking central stimulus, %	−59 (345)	4	−116 (192)	1.5	−231 (336)^c^	2	99 (371)	3	−254 (267)^c^	1.5	−398 (308)^c^	1
Mean distance from stimulus, pixels	7.8 (16.7)	4	4.0 (4.2)	2	15.8 (25.9)	2	−4.2 (15.5)^d^	2	17.8 (23.0)^c^^,^^d^	0.5	35.4 (33.8)^c^	1
Total correct, No.	−1.3 (2.4)	5	−1.0 (2.0)	4	−2.3 (3.9)	2	1.6 (6.3)^d^	0.5	1.1 (6.5)	8	−3.6 (5.6)	1
PASAT												
Total correct, No.	−7.8 (12.6)	5	−4.6 (5.6)	2	−15.4 (24.7)	1	3.3 (13.1)	2	−7.1 (13.0)	1.5	−21.8 (24.9)^c^	0.5
Reaction time correct, ms	−38.7 (107)	8	80.3 (119)^c^	2	84.9 (90.8)^c^	0.5	−43.8 (80.8)	8	86.9 (104)^c^	1.5	85.4 (162)^c^	1.5
Reaction time incorrect, ms	412.8 (476)	3	341.8 (591)	1.5	562.8 (782)	4	244.7 (614)	8	364.6 (375)	5	493.5 (616)	4
Physiological measures												
Heart rate, beats/min	−3.7 (8.5)	1.5	11.3 (10.4)	0.17	19.1 (17.1)^c^	0.17	−4.3 (6.1)	1	23.3 (11.8)^c^^,^^d^	0.17	26.8 (16.6)^c^	0.17
Diastolic blood pressure, mmHg	4.5 (7.4)	8	−4.8 (10.9)	4	4.0 (10.0)	0.5	2.6 (7.6)	8	40.6 (174.8)^d^	1.5	−4.8 (6.4)	4
Systolic blood pressure, mmHg	7.8 (25.0)	4	−10.1 (23.9)^c^	1	−4.1 (14.2)	2	−2.3 (12.5)	0.5	−7.7 (15.5)	0.5	−6.1 (12.2)	0.5

Abbreviations: DAT, divided attention task; DEQ, drug effect questionnaire; DSST, digit symbol substitution task; NA, not applicable (no change from baseline); PASAT, paced auditory serial addition task; THC, Δ9-Tetrahydrocannabinol.

^a^ Indicates time point at which peak effects occurred for each outcome.

^b^ Items for the DEQ were assessed using a visual analog scale with scores ranging from 0 (not at all) to 100 (extremely).

^c^ Significant difference from 0-mg condition within that route of administration (all *P* values <.025).

^d^ Significant difference between smoked and vaporized conditions at that THC dose (all *P* values <.05).

At the 10-mg dose, vaporized cannabis, compared with smoked cannabis, resulted in higher mean (SD) ratings of drug effect (69.5 [26.4]), dry mouth (61.8 [23.6]), and dry, red, and/or irritated eyes (19.2 [28.9]), and lower ratings of vigorous and/or motivated (−21.8 [29.4]) drug effects (all *P* values <.05) (Table and Figure 2). At the 25-mg vaporized dose, mean (SD) ratings of paranoid (17.4 [30.0]) were significantly higher compared with the 25-mg smoked dose (10.0 [22.0]) (*P* < .05). The magnitude of changes observed for most drug effects were qualitatively larger when cannabis was vaporized, compared with smoked, and drug effects were mostly dose orderly (eg, qualitatively larger changes from baseline observed at the 25-mg dose vs the 10-mg and/or 0-mg doses within the same inhalation method). The majority of observed drug effects peaked between within the first hour after cannabis administration was completed (Table). Notably, for both 25-mg doses, mean ratings for drug effect had not returned to baseline 6 hours after cannabis administration (mean [SD], smoked: 12.2 [5.9]; vaporized: 10.8 [4.4]) (Figure 2).

### Cognitive and Psychomotor Measures

**Figure 3.**
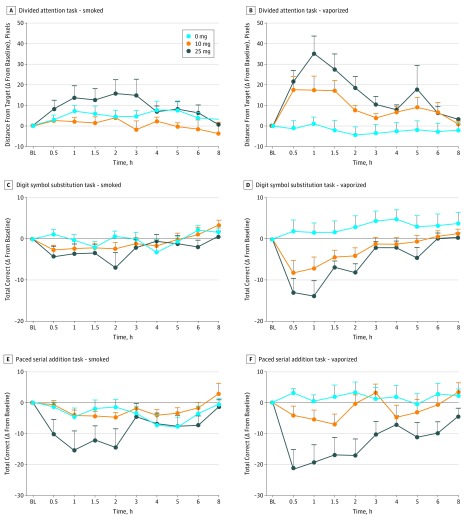
Mean Change From Baseline Scores for Average Distance From Central Stimulus From Divided Attention Task, Total Correct on Digit Symbol Substitution Task, and Total Correct on Paced Auditory Serial Addition Task A and B, Higher scores indicate poorer performance relative to baseline. C-F, Lower scores indicate worse performance relative to baseline. Error bars indicate SEM. All scores are shown over time and are displayed by Δ9-tetrahydrocannabinol dose and inhalation method. Horizontal axes are not accurate time scales and represent the time points at which cognitive and psychomotor performance was measured. BL indicates baseline time point; and Δ, difference or change.

Figure 3 displays primary outcomes from the DAT, PASAT, and DSST over time by inhalation method. Peak change in mean reaction time for correct responses on the PASAT was slower at both doses and inhalation methods (by ≥120 milliseconds) compared with placebo. The percentage of time spent accurately tracking the central stimulus on the DAT decreased from baseline following the 25-mg smoked cannabis dose (approximately 170%) and both vaporized cannabis doses (approximately 350% after 10 mg and approximately 500% after 25 mg) compared with placebo (all *P *values <.025). Both vaporized cannabis doses decreased the mean (SD) of number attempted (10 mg, −6.0 [10.0]; 25 mg, −10.0 [12.7]), number correct (10 mg, −8.3 [11.3]; 25 mg, −13.8 [14.9]), and percentage correct (10 mg, −7% [13]; 25 mg, −16% [28]) on the DSST and mean (SD) distance from the central stimulus (10 mg, 17.8 [23.0]; 25 mg, 35.4 [33.8]) on the DAT task, while the 25-mg vaporized dose reduced the total correct for the PASAT (mean [SD], −21.8 [24.9]) (all *P* values <.025; Table). Greater impairment was observed for vaporized cannabis compared with smoked cannabis on mean (SD) distance from the central stimulus for the DAT at 10 mg (17.8 [23.0] more pixels from baseline vs 4.0 [4.2] with 10 mg smoked), and for total attempted on the DSST at 25 mg (10 [12.7] fewer attempts from baseline with 25 mg vaporized vs 2.8 [6.4] fewer in smoked; all *P* values <.05). Cognitive and psychomotor deficits typically peaked between 30 and 60 minutes after cannabis administration and, in some instances, did not return to baseline for 6 to 8 hours (Figure 3).

### Physiological Measures

**Figure 4.**
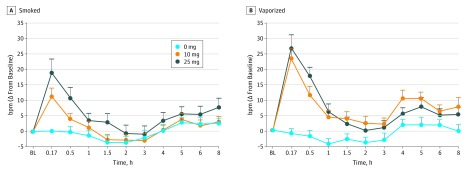
Mean Change From Baseline Score for Heart Rate Over Time, Displayed by Δ9-Tetrahydrocannabinol Dose for Smoked and Vaporized Conditions Error bars indicate SEM. Horizontal axes are not accurate time scales and represent the time points at which heart rate was measured. BL indicates baseline time point; bpm, beats per minute; and Δ, difference or change.

Observed increases from baseline for HR at the 25-mg smoked cannabis dose (mean [SD], 19.1 [17.1] beats/min) and both doses of vaporized cannabis (mean [SD], 10 mg, 23.3 [11.8]; 25 mg, 26.8 [16.6] beats/min) were significantly greater than placebo (all *P *values <.025) (Table). Systolic BP decreased significantly after the 10-mg smoked cannabis dose (mean [SD], −10.1 [23.9] mm Hg) compared with placebo but did not differ across vaporized doses (mean [SD]: 10 mg, −7.7 [15.5]; 25 mg, −6.1 [12.2] mm Hg). At the 10-mg dose, the magnitude of HR increase was significantly greater following vaporized cannabis (mean [SD], 23.3 [11.8] beats/min) compared with smoked cannabis (mean [SD], 11.3 [10.4] beats/min) (*P* < .05). On average, peak changes in HR occurred immediately (ie, at the 10-minute postdosing assessment point) and returned to baseline within 3 hours of cannabis administration (Figure 4).

### Whole-Blood THC Concentrations

Consistent with the pharmacodynamic outcomes, quantitative THC concentrations in whole blood were higher following vaporized vs smoked cannabis administration and demonstrated dose-orderly differences (eFigure in Supplement 2). For vaporized conditions, mean (SD) peak concentrations for THC in whole blood were 7.5 (5.5) ng/mL at the 10-mg dose and 14.4 (9.4) ng/mL at the 25-mg dose. For smoked cannabis conditions, mean (SD) peak THC concentrations were 3.8 (5.9) ng/mL at the 10-mg dose and 10.2 (12.4) ng/mL at the 25-mg dose. Blood THC concentrations peaked at the 10-minute postdosing time point and returned to 0 within 4 hours of dosing for all conditions.

### Correlations Between Whole-Blood THC Concentrations and Pharmacodynamic Profiles

For both smoked and vaporized cannabis, at both active doses, whole-blood THC concentrations were positively correlated with subjective ratings of drug effect (*r* > 0.37) and HR changes (*r* > 0.24). For the 25-mg vaporized cannabis dose, a significant negative correlation was observed between blood THC concentrations and the total correct responses on the DSST (*r* = −0.32). However, other indices of cognitive and psychomotor performance (ie, DAT and PASAT performance) were not significantly correlated with blood THC concentrations (eTable in Supplement 2).

### Adverse Events

Two participants vomited (1 after 25-mg THC vaporized inhalation and 1 after 25-mg THC smoked inhalation) and another experienced hallucinations^27^ after inhaling 25 mg of vaporized cannabis.

## Discussion

The current study provides a comprehensive evaluation of the acute effects associated with smoked and vaporized cannabis, at multiple THC doses, among healthy adults. Unlike prior controlled examinations of acute cannabis effects, participants in this study were not regular cannabis users. On average, participants last used cannabis about 1 year prior to enrollment, and none had used cannabis in the 30 days prior to enrollment. After inhaling smoked and vaporized cannabis containing 25 mg of THC, participants experienced pronounced drug effects, substantial impairment of cognitive and psychomotor functioning, and marked increases in HR. Notably, the highest dose of cannabis administered in this study (25 mg of THC: 0.19 g; 13.4% THC) is substantially smaller and has a lower THC concentration than what is typically contained in prerolled cannabis cigarettes available for purchase in cannabis dispensaries, which commonly contain roughly 1.0 g of cannabis with THC concentrations often exceeding 18%.^28^ Thus, individuals who initiate cannabis use can readily access products that contain far greater amounts of cannabis, with higher THC concentrations, than administered in this study. Regulatory and clinical entities should consider these results in decisions involving cannabis accessibility, dosing recommendations, and education for novice cannabis users.

In contrast to previous controlled comparisons of smoked and vaporized cannabis effects,^12,13,14^ in the current study, vaporized cannabis produced significantly greater subjective drug effects, cognitive and psychomotor impairment, and higher blood THC concentrations than the same doses of smoked cannabis. These discrepant results may be because procedures used in former studies enabled users to titrate their THC dose, whereas the current study required participants to self-administer a fixed amount of cannabis. Therefore, holding THC dose constant, vaporizers appear to be a more efficient cannabis and THC delivery method, likely because with traditional smoked preparations, more THC is lost as a result of pyrolysis (combustion) and/or sidestream smoke.^11^ Vendors and consumers of cannabis products should be aware that inhaling cannabis with a vaporizer could produce more pronounced drug effects and impairment than traditional smoking methods.

Interestingly, the time course of effects differed across outcome measures such that increases in blood THC concentrations and HR returned to baseline more rapidly than subjective drug effects and cognitive and psychomotor impairment. In several instances, cannabis-induced effects and/or impairments persisted for several hours after blood THC concentrations had fallen below the LOQ. Additionally, blood THC concentrations were only moderately correlated with subjective drug effects and weakly correlated, or not correlated at all, with cognitive and psychomotor performance. Collectively, findings from this study and others^16,29,30^ indicate that blood THC concentrations are not a valid indicator of a user’s intoxication and/or impairment from cannabis use and highlight the need to explore other biological and behavioral means of detecting acute cannabis impairment.

### Limitations

The current study has some noteworthy limitations. First, a limited range of doses, 1 type of cannabis (raw plant material: high THC/low CBD), and 1 type of vaporizer held at a fixed temperature were used. Future studies are needed to determine the generality of the effects found in the study to other forms of cannabis (eg, cannabis extracts and those with varied THC:CBD ratios). Additionally, vaporizer characteristics (eg, temperature and power output) could mediate THC delivery and should be explored further.^11^ Second, enrollment was limited to infrequent cannabis users. The extent to which chronic cannabis users or users who have a specific preference for vaporized or smoked cannabis differ on the outcomes examined in the study are unclear. Third, the small sample size of the current study precluded evaluation of participant characteristics (eg, genetics) that could influence acute cannabis effects.^31^

## Conclusions

In this study, participants experienced dose-orderly increases in subjective drug effects, cognitive and psychomotor impairment, acute cardiovascular effects, and blood THC concentrations following inhalation of smoked and vaporized cannabis. Notably, vaporized cannabis produced greater changes in study outcomes relative to smoked cannabis. As the legal cannabis marketplace continues to expand, future studies should further explore the effects of vaporizers and other novel methods for cannabis administration in users with different degrees of experience with cannabis, as the pharmacokinetic and pharmacodynamic profiles will likely differ substantially across products and users.

## Notes

Supplement 1. **Trial Protocol**

Supplement 2. **eFigure**. Mean Whole Blood Δ9-Tetrahydrocannabinol (THC) Concentration Over Time, Across Dose for (A) Smoked and (B) vaporized Conditions **eTable**. Correlations (Pearson *r*) Between Individual Change From Baseline Values for Whole Blood Δ9-tetrahydrocannabinol (THC) Levels and Pharmacodynamic Measures

Supplement 3. **Data Sharing Statement**
