# Error in Table Title

**DOI:** 10.1001/jamanetworkopen.2018.7241

**Published:** 2018-12-21

In the Original Investigation titled, “Acute Effects of Smoked and Vaporized Cannabis in Healthy Adults Who Infrequently Use Cannabis: A Crossover Trial,”^1^ published November 30, 2018, there was an error in the wording of the title of the Table. The title should have appeared as “Mean Peak Change From Baseline Values for Pharmacodynamic Measures by Inhalation Method and THC Dose.” This article has been corrected.^1^
