# Supplementary material for: Acute Effects of Smoked and Vaporized Cannabis in Healthy Adults Who Infrequently Use Cannabis: A Crossover Trial
Source: JAMA Netw Open. 2018 Nov 30;1(7):e184841. doi: 10.1001/jamanetworkopen.2018.4841 (PMC6324384; doi:10.1001/jamanetworkopen.2018.4841)
Supplement: Supplement 1. — Trial Protocol [file jamanetwopen-1-e184841-s001.pdf]

Study Protocol

Date: April/10/2016
Principal Investigator: Vandrey, Ryan
Application Number: IRB00035394

- **Use the section headings to write the eForm A, inserting the appropriate material in each. If a section is not applicable, leave heading in and insert N/A.**
- **When submitting eForm A (new or revised), enter the date submitted to the field at the top of eForm A.**

****

1. Abstract

a. Provide no more than a one page research abstract briefly stating the problem, the research hypothesis, and the importance of the research.

Understanding the pharmacokinetics of cannabis and comparative pharmacodynamics effects is important for a number of reasons. First, the pharmacokinetic profile of cannabis is essential to understanding and interpreting toxicology testing for detection of use. Detection of cannabis use is important for the conduct of controlled clinical trials of treating cannabis use disorders and for workplace and roadside drug testing programs. Establishing behavioral and subjective effect profiles in parallel with pharmacokinetics will be important for determining biological concentrations associated with intoxication/impairment and distinguishing between acute/recent use and residual levels of cannabinoids from long-term use. Such data is important for advising regulations related to driving under the influence, and can be used to reinforce early abstinence behavior in patients engaging in a quit attempt in a formal treatment setting.

In substance abuse treatment programs, contingency management (CM) is an effective, evidence-based, treatment based on the principles of behavior analysis. Patients trying to quit are provided rewards for providing objective evidence of drug abstinence. Typically, this is done using urine drug testing, and for substances like cocaine, tobacco, and opiates, this is very effective. Using CM for the treatment of cannabis use disorders is more difficult because cannabis remains detectable at high levels for much longer periods of time after use, and may last longer depending on the route of administration. Research is needed to determine whether biological matrices other than urine have a shorter window of detection following long-term chronic use but still register reliable “positive” results with recent use episodes. Research is also needed to evaluate whether there are differences in clearance based on the route of cannabis administration.

Workplace drug testing is practiced broadly in the United States in both the Federal Government and the private sector. More than 17 million people over age 18 were illicit drug users in 2007, according to the U.S. Department of Labor, and more than 75 percent were currently employed. Drug use can contribute to workplace accidents and cause an increase in absenteeism. Thus, it is clear that drug testing is necessary to maintain a safe, drug-free workplace. Although urine is the only biological specimen currently approved for use in the Federal workplace program, oral fluid is tested for drugs in the private sector, and there is interest in adding oral fluid as an alternate test matrix in the Federal Program. Prior to adopting oral fluid in the Federal Program, additional validity testing is required.

THC is reliably found in oral fluid following smoked cannabis and oral ingestion of extracted active constituents (e.g. dronabinol), but few studies have been conducted to assess the pharmacokinetic and pharmacodynamics of different doses of orally consumed intact cannabis (e.g., cannabis-containing brownies) on drug test results, and no published studies have characterized oral fluid cannabinoid levels following vaporization of cannabis. Careful analyses of these parameters is required to determine the level and duration of cannabinoid detection after oral consumption and vaporization, relative to smoked cannabis via oral fluid testing prior to implementation in workplace settings.

The present study will be conducted in 3 phases. In Phase 1, we will evaluate the detection of cannabinoids in oral fluid, plasma, hair, and urine for up to 9 days following consumption of a brownie containing one of three possible doses of cannabis containing 10mg, 25mg, or 50mg THC. This design will allow us to assess the effects of cannabis dose on drug detection across a range of biological matrices. In Phase 2, we will conduct a dose-effect evaluation of the subjective, behavioral, and cognitive performance effects of oral cannabis ingestion among study participants at each active dose and following placebo using a within-subject crossover design. In Phase 3, we will evaluate the effects of smoked and vaporized cannabis containing 0mg (placebo), 10mg and 25mg THC doses for direct comparison with the pharmacokinetic and pharmacodynamic outcomes obtained in Phase 2. The result will be a comparative pharmacology and toxicology data set across the most common routes of cannabis administration. This data will be invaluable for informing basic behavioral pharmacology of cannabis, cannabis policy and forensic interpretation of biological specimen analyses as they relate to behavioral outcomes.

Presently, there is considerable interest in inclusion of oral fluid as a test matrix in the US Federal workplace drug-testing program. This study will provide important information on how long various doses of orally consumed cannabis can trigger positive oral fluid (and other biological matrices) drug tests. Additionally, the parallel assessment of cognitive performance and quantitative toxicology testing may help inform guidelines for assessing impairment in suspected "drugged driving" cases involving oral cannabis use.

2. **Objectives** (include all primary and secondary objectives)

Objective 1: Examine the time course of the effects of orally ingested cannabis on results of drug tests using "native" oral fluid, urine, blood and hair specimens collected from individuals without any recent (past 3 months) cannabis use.

Objective 2: Examine the influence of dose on the pharmacokinetics and pharmacodynamics of orally ingested cannabis, and on results of drug tests using "native" oral fluid, urine, blood and hair specimens.

Objective 3: Examine the dose effects of oral cannabis on subjective, physiological, and cognitive performance assessments, and correlate these outcomes with quantitative biomarkers in multiple biological matrices.

Objective 4: Determine the comparative pharmacokinetics and pharmacodynamics of oral, smoked, and vaporized cannabis.

3. **Background** (briefly describe pre-clinical and clinical data, current experience with procedures, drug or device, and any other relevant information to justify the research)

Understanding the pharmacokinetics of cannabis and comparative pharmacodynamics effects is important for a number of reasons. First, the pharmacokinetic profile of cannabis is essential to understanding and interpreting toxicology testing for detection of use. Detection of cannabis use is important for the conduct of controlled clinical trials of treating cannabis use disorders and for workplace and roadside drug testing programs. Establishing behavioral and subjective effect profiles in parallel with pharmacokinetics will be important for determining biological concentrations associated with intoxication/impairment and distinguishing between acute/recent use and residual levels of cannabinoids from long-term use. Such data is important for advising regulations related to driving under the influence, and can be used to reinforce early abstinence behavior in patients engaging in a quit attempt in a formal treatment setting.

In substance abuse treatment programs, contingency management (CM) is an effective, evidence-based, treatment based on the principles of behavior analysis. Patients trying to quit are provided rewards for providing objective evidence of drug abstinence. Typically, this is done using urine drug testing, and for substances like cocaine, tobacco, and opiates, this is very effective. Using CM for the treatment of

cannabis use disorders is more difficult because cannabis remains detectable at high levels for much longer periods of time after use, and may last longer depending on the route of administration. Research is needed to determine whether biological matrices other than urine have a shorter window of detection following long-term chronic use, but still register reliable “positive” results with recent use episodes.

Workplace drug testing is practiced broadly in the United States in both the Federal Government and the private sector. More than 17 million people over age 18 were illicit drug users in 2007, according to the U.S. Department of Labor, and more than 75 percent were currently employed. Drug use can contribute to workplace accidents and cause an increase in absenteeism. Thus, it is clear that drug testing is necessary to maintain a safe, drug-free workplace. Although urine is the only biological specimen currently approved for use in the Federal workplace program, oral fluid is tested for drugs in the private sector. In addition, there is interest in adding oral fluid as an alternate test matrix in the Federal Program.

Although urine has been the predominant specimen of choice for conducting drug tests, it has clearly defined collection weaknesses that have been recognized since its first use. Drug abusers have found ways to foil the drug test in a variety of innovative ways. Prior to showing up for a drug test, drug abusers know that by “water-loading” they may escape detection by providing a highly dilute specimen thereby lowering drug concentrations below detection thresholds (1). A second dilution method is simply adding fluid to the specimen during collection. However, laboratories have become adept at detecting a “dilute” specimen; therefore, many drug abusers take additional precautions to improve their chances of escaping detection. Many commercial products are now available that can be added to urine during collection, thereby adulterating the specimen and producing a false negative result when tested.

The use of oral fluid as a test matrix may overcome some of the weaknesses found in the urine drug testing program. Oral fluid is primarily saliva and is easily collected with an absorptive device placed in the mouth or collection of “spit” in a sterile container. Collection takes only a few minutes and the collector observes the entire process from start to finish, thus eliminating attempts by the donor to beat the test. Oral fluid testing preserves individual privacy while allowing for direct observation without embarrassment (2). If an additional specimen is desired, either simultaneous collection or sequential collection can be part of the routine procedure. Oral fluid collections also eliminate gender collection problems and “shy bladder” issues associated with urine collection.

Salivary glands on the cheek and under the tongue supply the major fluid component to oral fluid. These glands have high blood flow; consequently drugs migrate rapidly from blood to salivary glands and appear in saliva within minutes of drug administration (3). For many of the major drugs of abuse, clinical studies have demonstrated parallel drug/metabolite relationships between oral fluid and blood. Thus, oral fluid serves as a “window” into the body for most drugs. Detection times for drugs in oral fluid tend to be similar or longer than detection times in blood but generally shorter than in urine. A review of detection times of drugs of abuse in blood, urine and oral fluid, concluded that drugs can be detected for 5 to 48 hours in oral fluid as compared to 1.5 to 4 days in urine following a single drug dose and for a week or longer following chronic drug use (4).

Hair is another type of biological matrix that can be tested for drugs. Head hair grows at an average rate of 1.3 cm/month although there is some variation according to sex, age and ethnicity. There are multiple possible pathways for drug incorporation into hair including: 1) passive diffusion from blood into the hair follicle; 2) excretion onto the surface of hair from sweat and sebum; 3) passage from skin to hair; and 4) from external contamination. Drug entering hair via blood from the capillary plexus of the follicle is not detectable by standard hair cutting methods until hair grows to the skin surface. In controlled dosing studies with cocaine and codeine, these drugs were detectable in “unwashed” human head hair approximately eight days after the first drug administration (5). Environmental contamination of hair also can occur and confounds interpretation of drug tests involving hair.

Cannabis (marijuana) is the most commonly used illicit drug worldwide. Rates of use have been increasing in recent years, corresponding with greater social acceptability, decreased perceived harm, increased use of “medical marijuana”, and legalization in some jurisdictions. The principal psychoactive

constituent of cannabis is delta-9-tetrahydrocannabinol (THC). THC also is found in pharmaceutical preparations, e.g., dronabinol, a light yellow resinous oil insoluble in water and formulated in sesame oil. The primary route of administration of cannabis is by smoking, but ingestion of cannabis products as foodstuffs is not uncommon. THC appears rapidly in plasma following the smoking of marijuana (6). Oral ingestion generally produces lower blood concentrations and delays in time to peak effects (7,8). The highly lipophilic nature of THC allows rapid tissue uptake with concomitant decreases in plasma. THC appears to be released slowly from tissue resulting in a prolonged half-life of THC and metabolites. THC is metabolized by hydroxylation to an active metabolite, 11-hydroxy-THC, which in turn, is oxidized to 11-nor-9-carboxy-Δ9-tetrahydrocannabinol (THCCOOH). THCCOOH is excreted in urine as the water-soluble glucuronic acid conjugate.

THC is found in oral fluid following smoked (9,10) and oral ingestion (11) of cannabis. THCCOOH is also found in oral fluid at very low concentrations. Based on evidence to date, it appears that THC is present in oral fluid primarily as a result of deposition in the oral cavity, rather than from transfer from blood (12). Following ingestion of hemp oil liquid containing THC and capsules of dronabinol, positive oral fluid tests for THC did not occur (13), but it is unknown whether or not deposition occurs after consuming cannabis plant material orally, such as via a cannabis brownie. Also lacking in the published literature are careful studies in which the dose effects of orally administered and vaporized cannabis are characterized in oral fluid, including a full time course evaluation.

Evaluation of dose effects is important because the potency of cannabis has risen steadily over the last 15 years. ElSohly, et al. reported the mean Δ^9-tetrahydrocannabinol (THC) of all confiscated cannabis preparations to be 8.8% in 2008. Currently, the average potency is about 10% THC, compared to 1983 when it was less than 4%. Consequently, many of the older clinical studies that formed the knowledge basis for interpretation of drug tests are outdated and need to be repeated with the higher potency cannabis that is representative of current use. The stronger cannabis is of particular concern because of the higher THC concentrations. While experienced cannabis users may limit their intake of potent cannabis, young and inexperienced users may not moderate their intake and possibly suffer from dysphoria, paranoia, irritability and other negative effects. This may be particularly true with orally consumed cannabis since it is much more difficult to self-titrate to a desired dose, as peak effects occur a considerable amount of time after oral administration.

Presently, there is considerable interest in inclusion of oral fluid as a test matrix in the US Federal workplace drug-testing program, and as an alternative to urine drug testing in clinics conducting controlled research in the treatment of cannabis use disorders. This study will provide important information on the time course of cannabis triggering positive oral fluid (and other biological matrices) drug test results following oral consumption, and inhalation of smoked and vaporized cannabis. Additionally, the parallel assessment of cognitive performance and quantitative toxicology testing may help inform guidelines for assessing impairment in suspected “drugged driving” cases involving suspected cannabis use across different routes of administration.

4. Study Procedures

a. Study design, including the sequence and timing of study procedures (distinguish research procedures from those that are part of routine care).

<u>Protocol Overview.</u> The proposed study will be conducted at the Johns Hopkins Behavioral Pharmacology Research Unit (BPRU) and the Johns Hopkins Bayview Clinical Research Unit (CRU). Participants will complete the study in 3 separate phases. Phase 1 will be a 9-day session, consisting of a 6-day (130 hour) residential stay at the CRU and a 3-day outpatient period. The purpose of Phase 1 is to fully characterize the pharmacokinetics of oral cannabis at 3 different active cannabis doses (conditions 1-3 below) in “native” oral fluid, whole blood, urine and hair. Phase 2 will consist of 4 outpatient sessions conducted at the BPRU, each separated by at least one week. The purpose of Phase 2 is to characterize the pharmacodynamic dose-effects of oral cannabis (conditions 1-4 below) using a placebo controlled within-subject crossover design.

- Condition 1: A chocolate brownie containing approximately 10mg of THC (prepared with 100mg of cannabis that contains approximately 10.0% THC).
- Condition 2: A chocolate brownie containing approximately 25mg of THC (prepared with 250mg of cannabis that contains approximately 10.0% THC).
- Condition 3: A chocolate brownie containing approximately 50mg of THC (prepared with 500mg of cannabis that contains approximately 10.0% THC).
- Condition 4 (Placebo): A chocolate brownie containing 0mg of THC (prepared with 250mg of cannabis from which THC and other psychoactive cannabinoids have been extracted).

Phase 3 will consist of 6 outpatient sessions conducted at the BPRU, each separated by 1 week, and following the same protocol as Phase 2. In Phase 3, cannabis plant material containing 0 mg, 10mg, and 25 mg will be smoked and vaporized by study participants.

For Study Phase 1, research volunteers will be recruited until each active dose condition (Conditions 1-3) is administered to 6 unique study participants (Total N = 18; N = 6 per dose condition). Immediately before (baseline) and following each exposure, a battery of assessments including biological fluid collection and testing, subjective questionnaire administration, and performance testing will be conducted for all study participants. Post-exposure testing will be conducted in two phases: a 6-day (130 hour) residential stay, and a 3-day outpatient period, for a total of 9 days. Participants who drop out of the study prior to completion of the residential study period will be considered “incomplete” and replaced. Data from Phase 1 will be used to complete Objectives 1 and 2 of the study.

For Study Phase 2, research volunteers will be recruited until up to 18 participants have completed each of the four study sessions (received Conditions 1-4). Participants who successfully complete Phase 1 will be invited to complete Phase 2. In Phase 2, participants will complete four outpatient study sessions, lasting approximately 10 hours each, separated by at least one week. Oral cannabis doses will be administered in a randomized order so that a full dose-ranging crossover is achieved for each participant. Similar to Phase 1, a battery of assessments including biological fluid (oral fluid, whole blood and urine) collection and testing, subjective questionnaire administration, and performance testing will be conducted at baseline and for 8 hours post-drug administration for all study participants. A wireless wearable electrocardiogram monitor will be used to continuously record cardiac signals for assessment of autonomic signatures of cannabis in each session. Phase 2 will be used to complete Objective #3 of the study.

For Study Phase 3, research volunteers will be recruited until up to 18 participants have completed each of the six study sessions. Participants who successfully complete Phase 1 and/or Phase 2 will be invited to complete Phase 3. In Phase 3, participants will complete six outpatient study sessions, lasting approximately 10 hours each, separated by at least one week. Sessions will be blocked such that the three doses of cannabis will be smoked in sequential sessions and vaporized in sequential sessions. The order of smoked versus vaporized administration will be counterbalanced across participants. Doses within each route of administration block will be administered in a double blind and random order. Route of administration will not be blinded. Similar to Phases 1 and 2, a battery of assessments including biological fluid (oral fluid, whole blood and urine) collection and testing, subjective questionnaire administration, and performance testing will be conducted at baseline and for 8 hours post-drug administration for all study participants. A wireless wearable electrocardiogram monitor will be used to continuously record cardiac signals for assessment of autonomic signatures of cannabis in each session. Phase 3 will be used to complete Objective #4 of the study.

There are several reasons to conduct the study in 3 separate study phases. First, to achieve the pharmacokinetic objectives (Objectives 1 and 2), a lengthy residential stay (6 days) and total period of assessment (9 days) is required, which is associated with significant cost and participant burden. The residential stay will ensure that participants are not exposed to additional cannabis during the period of evaluation, and the total time of 9-days will ensure that we get the full time course of detection across

biological matrices. Because we are only recruiting participants who have not used cannabis for at least 3 months at the time of study enrollment (and have no detectable cannabinoids in bio-specimens), there would be no value in conducting a 9-day pharmacokinetic evaluation of participants following administration of placebo. In order to properly achieve the pharmacodynamic objectives (Objectives 3 and 4), on the other hand, placebo drug administration is required to properly interpret drug effects and eliminate effects of expectancy and non-pharmacological factors (e.g. fatigue, hunger, etc.). In addition, due to individual variability in pharmacodynamics outcomes (e.g. subjective ratings of drug effects and cognitive ability), statistical power to detect differences between doses is significantly increased when using a within-subjects design versus a between subjects design. However, pharmacodynamic assessments require a shorter period of evaluation compared with complete pharmacokinetic evaluation because the time course of intoxication is approximately 4-6 hours, but biological detection may last for several days. Thus, the pharmacodynamic evaluation sessions can be conducted repeatedly within a much shorter time frame and with less time between sessions.

Participants. We will recruit and consent up to 40 research volunteers in order to obtain 18 study completers for each study phase (Total of 120 volunteers and 54 study completers total). We anticipate that some participants may drop out of the study before completion. It is estimated that we will need to enroll 24 participants to achieve 18 completers in each study phase. Those who complete the 6-day inpatient study phase will be considered evaluable for Phase 1, those who complete all 4 sessions of Phase 2, and those who complete at least all 3 doses of one route of administration in Phase 3 will be considered evaluable. We anticipate that most Phase 1 study completers will elect to complete Phase 2, and most participants who complete Phase 2 will elect to complete Phase 3, but, if needed, separate individuals will be screened to participate only in Phase 2 or Phase 3 in order to reach the completion target. Volunteers who drop out prior to completing the inpatient stay in Phase 1 or who do not complete the required number of sessions in Phase 2 will be replaced.

The target demographic for study participation are healthy adults who: 1) have a history of intentionally consuming cannabis, 2) have not used cannabis in the past month (desire is to have participants free of cannabinoids in biological matrices at the time of initial drug administration), and 3) who are not currently dependent on or seeking treatment for use of cannabis or other psychoactive drugs.

Participant recruitment. Participants will be recruited into the study via media advertising (e.g. newspaper, internet) and word-of-mouth communication. Advertisements will seek healthy adults who occasionally use cannabis and are not currently trying to quit. Interested participants will receive a brief screening over the telephone and will be scheduled for an in-person assessment if they meet initial eligibility criteria.

Prior to the in-person assessment, written informed consent to administer the assessment will be obtained. The assessment will be comprised of interviews and self-report surveys that provide participant information regarding health status including physical, mental health, and recreational drug use history. Urine specimens will be obtained and tested for evidence of recent use of commonly abused drugs. Participants must test negative for metabolites of THC, the primary psychoactive constituent of cannabis, and self report no cannabis use during the prior month. Participants must provide a government-issued photo ID confirming they are 18-45 years old, report prior use of cannabis at least once in their lifetime, and report no allergies to any of the ingredients used to prepare the brownies (e.g., chocolate, egg, wheat, etc.). Study participants will also undergo a physical exam including clinical chemistry, hematology, serology, and serum pregnancy test (females only). An electrocardiogram (EKG) reading will be obtained and reviewed by a physician or nurse practitioner to assess current cardiovascular health. Additionally, participants will be required to demonstrate that they can expectorate at least 3 mL of “native” oral fluid (saliva) over a 5-minute period. Those who appear eligible for participation will receive training on the study assessment measures (e.g. exposure to subjective questionnaires and cognitive performance tasks). Participants who successfully complete Phase 1 of the study will be invited to participate in Phase 2, and those who complete Phase 1 and/or 2 will be invited to participate in Phase 3. Separate written informed consent to participate will be obtained for all study phases. If more than 60 days passes between participation in Phase 1 and Phase 2, Phase 2 and Phase 3, or between any individual sessions in Phases 2 or 3, participants will be re-screened for eligibility to continue.

Experimental Session Procedures. The same general study procedures will be used for all 3 study phases for ease of comparison. For all study sessions, participants will be scheduled to arrive early in the morning on the day of cannabis exposure. All participants will complete a breath alcohol test on arrival. Participants with a positive BAL will be immediately discharged from the study. Urine drug and pregnancy testing will then be conducted for all participants to test for evidence of recent illicit drug use (e.g. cannabis, cocaine, opioids) and pregnancy. Volunteers must have negative urine drug screens on the day of the cannabis exposure session to participate. During Phases 2 and 3, the one-week minimum interval between sessions should be sufficient for eliminating cannabinoids between test days in such a manner that positive urine drug screens from the prior study exposure is not a concern. Participants will be fed a standardized low fat breakfast (e.g. toast and jam) each morning prior to cannabis administration. Participants will wear a cardiac signal recording device on their chest.

Baseline Assessments. Prior to cannabis administration, baseline oral fluid, urine, and blood and will be obtained from all participants. A baseline hair specimen will also be obtained in Phase 1. Baseline cardiovascular, subjective, and performance assessments will also be conducted (see below for details), and the TLFB will be conducted to record substance use since the last study visit (intake assessment or prior to experimental session). Concomitant medications**,** including vitamins and herbal supplements taken within 14 days prior to experimental session will be recorded. Changes in medication occurring between the screening assessment and experimental session will be reviewed by a study investigator and medical staff prior to cannabis administration to ensure the volunteer is still eligible to participate.

Cannabis Exposure. After baseline assessments are completed, participants will self-administer cannabis measured to produce targeted THC doses of 0, 10mg, 25mg, or 50mg. In Phase 1, participants will be randomized to receive one of the 3 active cannabis doses (10, 25, or 50mg THC) administered in cannabis containing brownies. In Phase 2, participants will receive each of the 4 doses in a cannabis containing brownie (one per session) in a randomized order. In Phase 3, dried cannabis will be smoked using a small commercial pipe. Placebo (<1% THC) and active (10% THC) cannabis will be prepared by the BPRU pharmacy such that the same volume of cannabis will deliver 0 mg, 10 mg, or 25 mg of THC across the three dosing sessions (placebo and active cannabis will be blended together to create each dose). Using the same cannabis preparations, dried cannabis will also be administered via commercial vaporizer. We will use The Volcano (Storz and Bickel, GmbH & Company (Oakland, CA), a vaporizer that has become the standard for controlled cannabis research involving vaporization. The Volcano is an approved medical device in several countries including Canada and Germany) and meets U.S. regulatory standards for an electric medical device. It has been demonstrated to reliably deliver doses of THC from dried cannabis. Either a pipe or vaporizer, filled by the pharmacy with the appropriate dose, will be dispensed to study participants for self-administration. On smoking days, they will be instructed to smoke the entire contents of the pipe. For vaporization sessions, The Volcano will be used to vaporize the entire dose into a balloon, the contents of which will be inhaled completely by the study participant. Cannabis brownies will be prepared using individual baking trays for each study participant. Baking will occur using a small oven located in the BPRU pharmacy and a commercial brownie mix (e.g. Duncan Hines Double Fudge Brownie Mix). The mix will be prepared according to manufacturer's instructions, with a measured dose of finely ground cannabis added to a portion the brownie batter mixture sufficient to make one cannabis brownie. The left over brownie mix will be discarded. Preparing brownies individually in this manner will allow us to ensure that cannabis doses are exact (vs. preparing multiple brownies at once where some error in plant matter distribution throughout the batter mixture is a possibility). Dose assignment will be conducted by the BPRU pharmacy in a manner that most balances gender, BMI, and race/ethnicity across dose conditions in Phase 1. This is not a concern for Phases 2 or 3 because they are within-subject crossover studies. Study participants and research staff will be blind to dose assignment. Participants will be provided with cannabis and drinking water approximately one hour after finishing their standardized low fat breakfast, and will be instructed that they need to self-administer the cannabis (eat brownie or inhale smoked/vaporized plant material) within 5 minutes. The conclusion of cannabis consumption will be considered the “0 hour” by which remaining protocol assessments will be scheduled. Cannabis smoking and vaporization will occur in a specially ventilated room in the BPRU

designed for the conduct of research with smoked/inhaled drugs that minimizes staff exposure to second-hand smoke/vapor.

For the remainder of the study, participants will complete a battery of assessments that includes biological specimen collection, subjective ratings on computerized questionnaires and cognitive performance. The period of evaluation for Phase 1 will be a single session lasting 9 consecutive days (6 days residential, 3 days outpatient). The period of evaluation for Phases 2 and 3 will be 4 and 6 outpatient sessions respectively, each lasing approximately 10 hours and separated by at least 7 days. Use of tobacco products will not be allowed during the study. Study participants who regularly use tobacco products will be provided nicotine patch upon request.

Phase 1 Inpatient Discharge. During Phase 1, participants will be discharged after completing final residential assessments (130 hours post-exposure). This is well past the time that any intoxicating effects of cannabis will have subsided (intoxication following oral cannabis/THC rarely exceeds a 6 hour time course). In cases in which a study participant indicates the desire to be discharged from the study early, research staff will review the self-reported rating of “drug effect” on the most recent subjective drug effect assessment and conduct a brief interview with the participant prior to discharge. If the participant reports a drug effect or exhibits behavior indicative of impairment/intoxication, the participant will be asked to remain at the BPRU/CRU until the drug effects subside and they can pass a field sobriety test. Taxi transportation home will be provided upon request or at the discretion of the research staff.

Phase 1 Outpatient Observation Period. Following discharge from the CRU, participants will be asked to return to BPRU/CRU once per day on 3 consecutive days in order to provide one urine and one oral fluid specimen at each visit.

Phase 2 and 3 Outpatient Discharge. Participants will be discharged after completing final assessments (approximately 8 hours post-exposure). This is expected to exceed the time course of intoxicating effects (intoxication following oral cannabis/THC rarely exceeds a 6 hour time course and inhaled cannabis effects usually resolve in 3-4 hours). If a study participant indicates the desire to be discharged from the study early, BPRU medical staff will review the self-reported rating of “drug effect” on the most recent subjective drug effect assessment and conduct a brief interview with the participant prior to discharge. If the participant reports a drug effect or exhibits behavior indicative of impairment/intoxication, the participant will be asked to remain at the BPRU until the drug effect subsides and they can pass a field sobriety test. All participants will be instructed not to drive home and to make alternative transportation arrangements. If a participant fails to arrange a ride, taxi transportation home will be coordinated by study staff and provided free of charge.

<u>Study Measures</u>. A battery of measures will be used to assess participant characteristics and drug effects during the study.

Screening. During the laboratory screening assessment, a battery of measures will be administered to collect background demographic data (age, gender, self-reported race and ethnicity, height, and weight) and to determine study eligibility (e.g. Medical History Interview, Drug-History Questionnaire, and Time-line Follow-Back (TLFB)). A physical examination will be performed on each subject during the Screening Visit. All major organ systems, including head, eyes, ears, nose, and throat (HEENT); cardiovascular system; lungs; abdomen (liver/spleen); extremities; skin; central nervous system (CNS); musculoskeletal system, and general appearance. A 12-lead EKG will be conducted to ascertain cardiovascular health and biological specimens will be tested for routine clinical chemistry, hematology, serology, serum pregnancy test (females only), and for evidence of recent illicit drug use.

Experimental Sessions. In Phase 1, vital signs **(**heart rate, systolic blood pressure (SBP), diastolic blood pressure (DBP)) will be measured in the seated position at baseline, 10 minutes after ingestion (time participants finish consuming brownie and rinse with water) and 0.5, 1, 1.5, 2, 3, 4, 5, 6, 8, 12, 22, 26, 30, 34, 46, 50, 54, 58, 70, 74, 78, 82, 94, 98, 102, 106, 118, 122, 126, and 130 hours post exposure. Data collection for Phases 2 and 3 will be identical to that for Phase 1 except that assessments will stop at 8

hours post exposure.

For Phase 1, blood sampling (10ml per specimen) will occur at baseline, 10 minutes after ingestion, and 0.5, 1, 1.5, 2, 3, 4, 5, 6, 8, 12, 22, 26, 30, 34, 46, 50, 54, 58, 70, 74, 78, 82, 94, 98, 102, 106, 118, 122, 126, and 130 hours post exposure. Data collection for Phases 2 and 3 will be identical to that for Phase 1 except that assessments will stop at 8 hours post exposure. Participants will have an indwelling intravenous catheter inserted prior to the start of the exposure session. Ten milliliters of blood will be collected by catheter at designated times into vacutainer tubes (gray top). Blood will be divided in half and transferred to two plastic cryotubes, labeled and stored frozen at -20 ºC until shipped frozen on dry ice to a designated laboratory for analysis. The maximum amount of blood to be collected during Phase 1 is 320ml, which is about two-thirds the amount typically collected during a routine blood donation (473ml). During Phases 2 and 3, 110ml of blood will be obtained per session, for a total of 440ml of blood across the 4 outpatient sessions in Phase 2 and 660ml of blood across 6 sessions in Phase 3. Due to the volume of blood collected, we will require a minimum of 30 days to pass between participation in study phases. This duration of time is adequate for blood to be replaced by healthy adults.

For Phase 1, oral fluid sampling will occur at baseline, 10 minutes after ingestion, and 0.5, 1, 1.5, 2, 3, 4, 5, 6, 8, 12, 22, 26, 30, 34, 46, 50, 54, 58, 70, 74, 78, 82, 94, 98, 102, 106, 118, 122, 126, and 130 hours post exposure. Three additional specimens will be obtained during the outpatient visits on Days 7, 8, and 9 after ingestion. Data collection for Phases 2 and 3 will be identical to that for Phase 1 except that assessments will stop at 8 hours post exposure. Collection of native oral fluid specimens will be performed by expectoration for a period of up to 5 minutes per sample into a labeled, 15-mL plastic centrifuge tube. No food or drink will be allowed during collection and for a period of 10 minutes prior to each scheduled collection. Each specimen will be sealed with a plastic screw cap and stored refrigerated until shipped to a designated laboratory.

A pre-dose urine sample (minimum of 60 mL) will be collected immediately preceding oral cannabis administration. Participants will be asked to attempt to void immediately after cannabis consumption and at 1, 2, 3, and 4 hrs post exposure. If participants need to urinate additionally over the first 4-hour period, their specimens will be labeled and stored as separate specimens. After the first four hours, pooled urine collections will be made over the following post-exposure intervals; 4-6 hours, 6-8 hours, 8-10 hours, 10-12 hours, 12-22 hours, 22-26 hours, 26-30 hours, and 30-34 hours, etc., up until the 130 hour time point for Phase 1 and stop after 8 hours post exposure for Phase 2 and 3 sessions. For Phase 1, three additional specimens will be obtained during the outpatient visits on Days 7, 8, and 9 after ingestion. During each collection period, either at designated times or as needed over the scheduled collection intervals, each participant will be asked to void their bladder into empty, clean, plastic collection containers, labeled with their identification number, date, and collection time. Each urine sample collected will be transferred to a labeled plastic pooling vessel (collection container provided with each collection kit) of adequate capacity for each scheduled collection interval. The plastic pooling vessels (with cumulative urine per interval) will be kept on ice or refrigerated by the study personnel during each collection interval. The volume of each collection pool will be recorded and 2 urine aliquots (minimum of 30 ml each; labeled #1 and #2) will be transferred to polypropylene bottles and frozen.

During Phase 1, participants will provide two hair specimens, one prior to cannabis ingestion and the second prior to discharge on the last residential study day (approximately hour 126). Hair specimens will be collected from the vertex area of the head. The collector holds strands of hair and cuts them with scissors near the root area (approximately 1-2 mm near the scalp). Approximately 80 mg of hair should be collected (80-100 hair strands). If the hair is sufficiently long (six centimeters (cm) or longer), the orientation of the cut hair should be maintained and identified. For example, if the hair is approximately 6 cm long, the collector should grasp the bundle of hair (80-100 hair strands) between their fingers and cut the hair bundle close to the scalp. The collector places the hair bundle in a piece of aluminum foil, folds the foil to secure the hair in the foil pouch, and marks the root end of the foil “root” and the other end “tip”. The foil pouch will be placed in a small plastic bag, labeled, and stored in the refrigerator until shipped to the analytical laboratory. For very curly hair, it may be difficult to maintain root/tip orientation. In these instances, the same collection procedures will be carried out with the exception of designation of “root/tip”

orientation of the sample. Hair samples will not be collected during Phase 2.

The BioPatch cardiac monitor (data sheet in supplementary information) is a FDA Class 2 Medical Device available by prescription or for research purposes by IRB approval (supplementary data sheet has been added to the eIRB application). The device will be attached to study participants via two disposable electrodes. Participants will be instructed on how and where to place the sensor on their chest and research staff will ensure it is properly placed. The sensor will be applied at baseline and will be worn continuously throughout each session. The device will log 1 lead electrocardiogram and 3-axis accelerometer data to memory. After the device is removed from the participant the data will be uploaded to a computer for analysis.

A 15-item Drug Effect Questionnaire will be used to obtain subjective ratings of intoxication. Individual items include ratings of drug effects (i.e. drug effect, good effect, bad effect) and behavioral/mood states often associated with marijuana intoxication (i.e. relaxed, paranoid, hungry/have munchies). Participants will rate each item using a 100mm visual analog scale (VAS) anchored with “not at all” on one end and “extremely” on the other. This questionnaire will be administered at baseline, immediately after exposure and 0.5, 1, 1.5, 2, 3, 4, 5, 6, and 8 hours post exposure. It is expected that all subjective drug effects will subside by Hour 8, but if a participant reports a drug effect beyond this time the questionnaire will continue to be administered at other time points until the participant no longer reports a drug effect.

Performance assessments will be conducted on aspects of cognitive/psychomotor functioning known to be sensitive to the acute effects of smoked marijuana and relevant to functioning in the workplace and/or in operating a motor vehicle or heavy machinery. All participants will be trained on the performance tasks to a stable baseline level during the screening session. Tasks include: 1) Divided Attention Task (DAT): Participants simultaneously perform two different simple tasks based on visual stimuli presented on a computer screen. Primary outcome is the accuracy with which they perform the two tasks; 2) Digit Symbol Substitution Task (DSST): Participants must hand type patterns presented to them on a computer screen for 90 seconds. Primary outcomes are accuracy and total number of patterns completed in the allotted time; and 3) a computerized version of the Paced Auditory Serial Addition Task (PASAT): Participants are provided a string of single digit numbers on the computer and must add the total of the prior to integers presented and respond by selecting the answer using the computer mouse on the screen. The primary outcome is a summed score of the number of correct trials during the task. Performance assessments will be completed at baseline and 1, 1.5, 2, 3, 4, 5, 6, and 8 hours post exposure.

b. Study duration and number of study visits required of research participants.

All participants will complete a visit for screening evaluation. Completion of Phase 1 requires a 6-day residential visit during the conduct of the experimental session and 3 outpatient study visits on three consecutive days following discharge from the 6-day residential visit. Phase 2 requires 4 outpatient drug administration sessions, lasting approximately 10 hours each, and spaced at least one week apart. Phase 3 requires 6 outpatient drug administration sessions, lasting approximately 10 hours each, and spaced at least one week apart. A minimum of one month must elapse between participation in Phases 1 and 2 and Phases 2 and 3.

c. Blinding, including justification for blinding or not blinding the trial, if applicable.

Cannabis dose assignment will be blinded in this study, but route of administration will not. It is standard procedure for appropriate scientific control in studies evaluating dose effects of psychoactive drugs to blind dose assignment. Blinding of route of administration is not necessary and would be difficult to manage in this study given the immediacy of effects via inhaled routes and the unpredictable delay in drug onset effects following oral administration.

d. Justification of why participants will not receive routine care or will have current therapy stopped.

Participants in this study will be healthy volunteers. Routine care for any medical illness that may arise during participation will not be affected.

e. Justification for inclusion of a placebo or non-treatment group.

A placebo dosing session will be included to help interpret active drug effects on pharmacodynamic outcomes. Placebo dosing provides a control for expectancy effects on subjective reports and cognitive performance as well as non-pharmacological factors such as fatigue, hunger, and learning effects on performance tasks. Placebo dosing is standard for research studies involving evaluation of acute drug effects.

f. Definition of treatment failure or participant removal criteria.

This is not a treatment study. Participants may quit participation at any time of their own volition. The study investigators will discharge study participants for failing to attend their scheduled session, failure to follow the protocol requirements, or for other reasons not known at this time.

g. Description of what happens to participants receiving therapy when study ends or if a participant's participation in the study ends prematurely.

This is not a treatment trial; there is no direct course of therapy related to the participant population being targeted. We are recruiting healthy adults with experience using cannabis and who are not seeking treatment for substance use problems. Should any report the desire for treatment they will be referred to appropriate community service centers. Premature termination of participation may result in the need to recruit additional research volunteers, but should have no impact on the study volunteer directly.

5. Inclusion/Exclusion Criteria

Participants will meet the following eligibility criteria:

Inclusion Criteria

1. Have provided written informed consent
2. Be between the ages of 18 and 45
3. Be in good general health based on a physical examination, medical history, vital signs, 12-lead ECG and screening urine and blood tests
4. Test negative for recent cannabis use in urine at the screening visit (confirmed by GC/MS laboratory test) and at clinic admission
5. Test negative for other drugs of abuse, including alcohol at the screening visit and at clinic admission
6. Demonstrate ability to expectorate 3-5 mL of "native" oral fluid over a 5-minute period
7. Not be pregnant or nursing (if female). All females must have a negative serum pregnancy test at the screening visit and a negative urine pregnancy test at clinic admission.
8. Have a body mass index (BMI) in the range of 19 to 36 kg/m2
9. Have head hair that is at least 4 cm (approximately one and a half inches) in length on the back of the head.
10. Blood pressure at Screening Visit does not exceed a systolic blood pressure (SBP) of 150 mmHg or a diastolic blood pressure (DBP) of 90 mmHg
11. Have no allergies to any of the ingredients used to prepare cannabis brownies (chocolate, eggs, wheat, etc.).

Exclusion Criteria

1. Non-medical use of psychoactive drugs other than, nicotine, alcohol, or caffeine 3 months prior to the Screening Visit;
2. History of or current evidence of significant medical or psychiatric illness judged by the investigator to put the participant at greater risk of experiencing an adverse event due to exposure or completion of other study procedures.

3. Use of an OTC, systemic or topical drug(s), herbal supplement(s), or vitamin(s) within 14 days of experimental sessions; which, in the opinion of the investigator or sponsor, will interfere with the study result or the safety of the subject.
4. Use of a prescription medication (with the exception of birth control prescriptions) within 14 days of experimental sessions; which, in the opinion of the investigator or sponsor, will interfere with the study result or the safety of the subject.
5. Use of hemp seeds or hemp oil in any form in the past 3 months.
6. Use of dronabinol (Marinol) within the past 6 months.
7. History of xerostomia (dry mouth), or the presence of mucositis, gum infection or bleeding, or other significant oral cavity disease or disorder that in the investigator's opinion may affect the collection of oral fluid samples.
8. History of clinically significant cardiac arrhythmias or vasospastic disease (e.g., Prinzmetal's angina).
9. Abnormal EKG result that in the investigator's opinion is clinically significant.
10. Enrolled in another clinical trial or have received any drug as part of a research study within 30 days prior to dosing.

6. Drugs/ Substances/ Devices

a. The rationale for choosing the drug and dose or for choosing the device to be used.

All cannabis will be obtained specifically for use in this study from the Federal Drug Supply System. During Phases 1 and 2 of the study, cannabis-containing brownies will be provided to and ingested by each study participant. Brownies will contain target THC doses of 0, 10, 25, or 50mg (i.e., 100, 250, or 500mg of cannabis material that is approximately 10% THC by volume; or 250mg cannabis for which THC and other cannabinoids has been extracted). During Phase 3 of the study, dried cannabis will be smoked and vaporized by study participants. In this study phase, THC doses of 0, 10, and 25mg will be administered via both routes. Smoking will occur using a hand-held pipe. Vaporization will be administered using a commercial vaporizer called The Volcano (Storz and Bickel, GmbH & Company (Oakland, CA). To preserve the blind, we will mix active and placebo cannabis so that the same amount of plant material is placed in the pipe and vaporizer at each session. The placebo dose will contain 250mg cannabis for which THC and other cannabinoids has been extracted. The 10mg THC dose will contain 100 mg cannabis with 10% THC and 150mg cannabis for which THC and other cannabinoids has been extracted. The 25mg dose will contain 250mg cannabis with 10% THC. Selection of doses was conducted to balance participant safety and tolerability based on our previous experience while maximizing the likelihood that we will administer doses that approximate current use patterns. The maximal dose (50mg) represents exposure to approximately half of a cannabis cigarette weighing 1 gram and containing 10% THC plant material. Research in our lab and others suggest that this is an average amount for a person to consume. Also, oral cannabis products sold in medical dispensaries often contain 50-100mg THC according to the package labels. A maximal dose of 25mg will be used for the smoked and vaporization routes of administration. This is because initial results from Phase 1 and 2 of the study suggest little difference between the 25 and 50mg doses on most study outcomes. Thus, we will eliminate one dose to reduce the total number of sessions required, and the highest dose will be eliminated because it would likely have an increased rate and severity of side effects compared with the 25mg THC dose.

Potential risks of consuming cannbis in the present study are stomach/gastrointestinal irritation and adverse effects associated with cannabis intoxication. We have considerable experience administering smoked cannabis and oral THC (dronabinol, Marinol) in our laboratory. In prior studies, we have safely administered acute THC doses up to 80mg and daily doses up to 240mg to daily cannabis users without significant adverse events. A minority of study participants reported nausea and discomfort following doses at or above 60mg. A colleague of ours, Dr. Joshua Lile at the University of Kentucky, has administered acute doses up to 90mg THC to weekly cannabis users. In that study, 5 of 7 participants tolerated all doses up to 90mg. Nausea and vomiting occurred in one participant following administration of the 30mg dose and in another participant following a 60mg dose. In a previously published study, Abrams and colleagues (2007) administered THC doses of 0, 7.6mg, 15.3mg, and 30.6mg via smoking and vaporization with The Volcano. Participants in that study were current cannabis users (at least once

in the past 30 days), but not heavy/daily users (maximum of 10 cannabis cigarettes or equivalent amount of plant material in the prior 30 days). All doses in that study were well tolerated. Thus, we believe that most study volunteers will be able to tolerate the proposed doses in the present study. If initial testing in this sample of less frequent cannabis users indicates difficulty then we will revise the proposed doses accordingly. Adverse events beyond nausea and vomiting are unlikely given the relatively safe pharmacological profile of THC (partial agonist), which has no history of being directly associated with fatalities. In cases where a participant experiences panic and or paranoid reactions, research staff will engage the person in relaxation exercises and will suspend research procedures until the volunteer has regained comfort. These types of effects are typically of short duration. In the case of an extreme adverse reaction, participants will be taken to the Johns Hopkins Bayview ER for treatment.

b. Justification and safety information if FDA approved drugs will be administered for non-FDA approved indications or if doses or routes of administration or participant populations are changed.

Not applicable to this protocol.

c. Justification and safety information if non-FDA approved drugs without an IND will be administered.

Not applicable to this protocol. An IND has been obtained for the administration of cannabis.

Study Statistics

a. Primary outcome variable.

The primary outcome variable for Phase 1 is the quantitative level of THC and its metabolites in different biological matrices (blood, oral fluid, urine, hair) from the three different doses of oral cannabis.

The primary outcome for Phase 2 is the subjective rating of “drug effect” on the DEQ.

The primary outcome variable for Phase 3 is the quantitative levels of THC and its metabolites in oral fluid, and subjective rating of “Drug Effect” on the DEQ.

b. Secondary outcome variables.

Secondary outcome variables include subjective drug effect and mood ratings on additional items of the DEQ, vital signs, and cognitive performance.

c. Statistical plan including sample size justification and interim data analysis.

The sample size estimation for Phase 1 was based on a confidence interval of 4 ng/mL (proposed oral fluid cutoff concentration), which requires a minimum of 6 exposed subjects. Oral fluid, blood, urine and hair specimens will be analyzed by an independent laboratory with validated analytical procedures that are specific and accurate for measurement of marijuana constituents and related metabolites. Data will be summarized by calculation of mean concentrations and standard deviation over time for each type of specimen.

The sample size estimate for Phases 2 and 3 was based on previous work in our laboratory evaluating dose effects of acute drug administration using a within-subjects design. A meta-analysis was previously conducted comparing the statistical power of 13 drug effect assessments from six dose-effect studies, with 14 participants each, evaluating a range of abused drugs in our laboratory (14). The analysis showed that average effect size for primary measures (i.e. subjective drug effect ratings, staff ratings and behavioral/cognitive performance measures) ranged from approximately 0.87 to 1.0. Based on this estimate of effect size, the proposed sample size of 18 should be adequate to assess the expected

effects. This sample selection methodology has been consistent in our long history of studies investigating dose-effects comparisons of different drugs, which have demonstrated excellent external validity and have become the FDA recommended standard for human abuse liability assessment. Subjective drug effect and mood ratings, vital signs and cognitive performance outcomes will be assessed using multiple regression analyses appropriate for repeated measures testing based on the final characteristics of the data set (e.g. normal distribution, skewness, kurtosis).

d. Early stopping rules.

The study will be stopped if new information is learned that indicates a serious risk to study participants.

7. Risks

a. Medical risks, listing all procedures, their major and minor risks and expected frequency.

Potential risks of cannabis exposure include dizziness, change in blood pressure, red or irritated eyes, drowsiness, easy laughing, euphoria, rapid heart rate, dry mouth, jitters, headache, nausea, vomiting, increased appetite, perceptual difficulties, memory lapse, hallucinations, confusion, depression, paranoid reaction, depersonalization, and rash. Additional potential risks of orally consuming cannabis are stomach/gastrointestinal irritation. We feel that the risk of serious adverse events related to cannabis exposure in this study is minimal, participants are experienced users and the doses we are administering are within the range by which most participants in our prior studies have had good tolerance to the drug.

Venous blood sampling may cause pain, tenderness, bruising, or bleeding at the needle puncture site. Some subjects may feel transient lightheadedness or dizziness, or lose consciousness (syncope), because of anxiety and vasovagal reaction. The only risk associated with oral fluid collection is dryness of the mouth.

A further risk is that participants may mistake the proposed studies as treatment or may delay treatment seeking in order to participate, although this is unlikely since we are targeting occasional users.

The Biopatch cardiac sensor should not be worn by subjects with a pacemaker or defibrillators.

Breech of confidentiality about self-reported drug use and biological tests indicating recent drug use is also a risk.

b. Steps taken to minimize the risks.

Participants are not a "vulnerable population" as defined by human subjects protection guidelines; that is, they are not minors, pregnant women, under legal coercion or restriction, or mentally impaired. They are competent adults who provide their voluntary informed consent. Participants will be recruited via media advertisements and posters that clearly state the nature and intent of the study. The consent process will inform the participant in detail of the procedures, time involvement, compensation, risk, and treatment options other than participation in our study. Particular emphasis will be given to providing information regarding the potential risks involved with taking the study drugs. Volunteers will also be instructed that they may withdraw from participation at any time without losing any of the compensation that they have earned to that point.

It is unlikely that any adverse event should arise that requires immediate medical or psychiatric treatment. However, in case of an adverse event, participants will be under the supervision of medical/nursing staff throughout the study. The medical and nursing staff at BPRU are trained in CPR and mobile emergency crash carts are available on the same corridor where all experimental procedures will be conducted. The research facility (BPRU) is located directly across the street from the Johns Hopkins Bayview Medical

Center Emergency Department, and, in case of an adverse event, participants will be taken for immediate care. The Principal Investigator will be immediately notified of any serious adverse events that arise.

If participants develop nausea or vomiting after consuming cannabis, study staff will assist the affected participant(s) appropriately and contact a study physician located in the building should the PI and/or study staff decide the participant would benefit from medical attention.

Blood collection risk will be minimized by performing venipuncture while participants are sitting down, and by having them remain under staff observation until it is clear that no acute adverse effects occur as a result of the procedure. The risk of infection is negligible because standard sterile technique will be used. Placement of indwelling venous catheters poses a risk of infection or thrombophlebitis, which increases with duration of placement. This risk is minimized by use of careful sterile technique, having nursing staff check the catheter at least once per shift (with prompt removal if there are clinically significant signs or symptoms such as tenderness, swelling, or redness), and limiting placement to 130 hours. Participants will also have the option of having catheter removed after Day 1 of the study when blood collection frequency is decreased. The risk of anemia is negligible because the total amount of blood to be collected within any 30-day period during the study is less than the amount (473 mL) collected within one hour during a single blood donation session. The amount of blood loss will be readily replaced without harm to study participants.

Individuals with a pace maker or defibrillator will not be given a heart rate monitor to wear during sessions. It is unlikely that any study volunteer with such devices would meet study eligibility requirements.

All advertisements and the informed consent process will clearly indicate that this research is designated only for those not seeking treatment, that participation is not a substitute for treatment, and that participation offers no clinical benefit. They will be clearly informed that they will be asked to ingest cannabis brownies during their participation. Any participant who expresses an interest in receiving immediate treatment for cannabis or other substance use will be referred to a community treatment clinic. If this occurs during the study, their participation in the study will be terminated. As previously described, participants will be instructed that should they withdraw from the study at any point to pursue treatment they will still be compensated for their participation up until that point in the study.

c. Plan for reporting unanticipated problems or study deviations.

The PI will also follow ICH regulations (detailed in *Clinical Safety Data Management, Definitions, and Standards for Expedited Reporting)* regarding reporting of adverse events and all study deviations to the IRB and study sponsor.

d. Legal risks such as the risks that would be associated with breach of confidentiality.

Participants' names will be recorded only on the screening, informed consent, and necessary medical and payment forms. Anonymous participant identification numbers will be used on all other forms and labeling of biological fluids and test results. All information gathered will be kept in locked research staff offices or file cabinets. All medical information obtained will be handled in accordance with HIPAA regulations. Only research staff will have access to participant records. The limits of confidentiality (e.g. suspected child abuse or neglect, or harm to self or others) will be discussed in detail with the participants during the informed consent process. To reduce the likelihood of patient records disclosure we have obtained a Certificate of Confidentiality.

e. Financial risks to the participants.

This study does not involve patients receiving treatment; therefore, the financial risks are minimal. Participants will be fairly compensated for their time and effort in complying with the study protocol.

8. Benefits

a. Description of the probable benefits for the participant and for society.

The primary benefit of the proposed research is in the knowledge gained regarding the relative biological, subjective and behavioral dose effects of exposure to cannabis administered orally, when smoked, or via vaporization. The knowledge will be used to advise the establishment of new drug testing guidelines across different biological matrices, and will inform the relevance of oral administration vs. inhalation on interpretation of workplace or roadside drug testing. The study will also extend the extant literature investigating the acute dose effects of cannabis, including subjective effects, cognitive performance, and their correlation with biological cannabinoid levels. Because we anticipate relatively minor risks to these cannabis experienced study participants, we feel that the proposed research has a positive risk benefit ratio.

9. Payment and Remuneration

a. Detail compensation for participants including possible total compensation, proposed bonus, and any proposed reductions or penalties for not completing the protocol.

All participants will be compensated $30 for completing the screening assessment. Compensation for full participation in Phase 1 is $2450, for Phase 2 is $1400, and Phase 3 is $2000. For participants who complete all 3 study phases $5880 of total earnings is possible. Compensation of this magnitude is appropriate given the length and nature of this study.

Screening Visit:	$30
Phase 1 Study Days 1-6:	$300/day ($1800 total)
Phase 1 Study Days 7-9:	$50/day ($150 total)
Phase 1 Completion Bonus:	$500
Phase 2 Sessions:	$300/session ($1200 total)
Phase 2 Completion Bonus:	$200
Phase 3 Sessions:	$300/session ($1800 total)
Phase 3 Completion Bonus:	$200
Total Compensation:	$5880

10. Costs

a. Detail costs of study procedure(s) or drug (s) or substance(s) to participants and identify who will pay for them.

The only direct costs to the participants will be their transportation to and from Bayview for each study visit. That cost has been factored into the compensation for participating.

Reference List

1. Cone,E.J., Lange,R., and Darwin,W.D. (1998), In vivo adulteration: Excess fluid ingestion causes false-negative marijuana and cocaine urine test results. *J.Anal.Toxicol.*, **22,** 460-473.

2. Cone,E.J. (2006), Oral fuid testing: New technology enables drug testing without embarrassment. *California Dental Association*, **34,** 311-315.

3. Cone,E.J. (1995), Pharmacokinetics and pharmacodynamics of cocaine. *J.Anal.Toxicol.*, **19,** 459-478.

4. Verstraete,A.G. (2004), Detection times of drugs of abuse in blood, urine, and oral fluid. *Ther.Drug Monit.*, **26,** 200-205.

5. Joseph,R.E., Jr., Hold,K.M., Wilkins,D.G., Rollins,D.E., and Cone,E.J. (1999), Drug testing with alternative matrices II. mechanisms of cocaine and codeine disposition in hair. *J.Anal.Toxicol.*, **23,** 396-408.

6. Huestis,M.A., Sampson,A.H., Holicky,B.J., Henningfield,J.E., and Cone,E.J. (1992), Characterization of the absorption phase of marijuana smoking. *Clin.Pharmacol.Ther.*, **52,** 31-41.

7. Alt,A. and Reinhardt,G. (1998), Positive cannabis results in urine and blood samples after consumption of hemp food products. *J.Anal.Toxicol.*, **22,** 80-81.

8. Cone,E.J., Johnson,R.E., Paul,B.D., Mell,L.D., and Mitchell,J. (1988), Marijuana-laced brownies: Behavioral effects, physiologic effects, and urinalysis in humans following ingestion. *J.Anal.Toxicol.*, **12,** 169-175.

9. Niedbala,R.S., Kardos,K.W., Fritch,E.F., Kunsman,K.P., Blum,K.A., Newland,G.A., Waga,J., Kurtz,L., Bronsgeest,M., and Cone,E.J. (2005), Passive cannabis smoke exposure and oral fluid testing. II. Two studies of extreme cannabis smoke exposure in a motor vehicle. *J.Anal.Toxicol.*, **29,** 607-615.

10. Niedbala,S., Kardos,K., Salamone,S., Fritch,D., Bronsgeest,M., and Cone,E.J. (2004), Passive cannabis smoke exposure and oral fluid testing. *J.Anal.Toxicol.*, **28,** 546-552.

11. Niedbala,R.S., Kardos,K.W., Fritch,D.F., Kardos,S., Fries,T., Waga,J., Robb,J., and Cone,E.J. (2001), Detection of marijuana use by oral fluid and urine analysis following single-dose administration of smoked and oral marijuana. *J.Anal.Toxicol.*, **25,** 289-303.

12. Huestis,M.A. and Cone,E.J. (2004), Relationship of delta-9-tetrahydrocannabinol in oral fluid to plasma after controlled administration of smoked cannabis. *J.Anal.Toxicol.*, **28,** 394-399.

13. Darwin,W.D., Gustafson,R.A., Sklerov,J., Fritch,D., Jacobs,A., Blum,K., Niedbala,S., Moolchan,E.T., and Huestis,M.A. (2004): Cannabinoid analysis of oral fluid collected from clinical subjects following oral tetrahydrocannabinol (THC) administration (hemp oil and dronabinol). *2004 Joint SOFT/TIAFT Meeting, Washington, DC*, August 30-September 3, 2004:300(Abstract)

14. Felch, L.J., M.E. Di Marino, and R.R. Griffiths, *A meta-analysis of psychomotor, subject- and observer-rated effects in abuse liability studies of anxiolytics*, in *In L.S. Harris (Ed.) Problems of Drug Dependence, 1995 (pp.188)*. 1996, Government Printing Office: Washington, DC.

15. Abrams, D.I., Vizoso, H.P., Shade, S.B., Jay, C., Kelly, M.E., and Benowitz, N.L. (2007). Vaporization as a Smokeless Cannabis Delivery System: A Pilot Study. *Clin Pharmacol Ther. 82, 572-578.*
