# Supplementary material for: Acute Effects of Smoked and Vaporized Cannabis in Healthy Adults Who Infrequently Use Cannabis: A Crossover Trial
Source: JAMA Netw Open. 2018 Nov 30;1(7):e184841. doi: 10.1001/jamanetworkopen.2018.4841 (PMC6324384; doi:10.1001/jamanetworkopen.2018.4841)
Supplement: Supplement 2. — eFigure. Mean Whole Blood Δ9-Tetrahydrocannabinol (THC) Concentration Over Time, Across Dose for (A) Smoked and (B) vaporized Conditions eTable. Correlations (Pearson r) Between Individual Change From Baseline Values for Whole Blood Δ9-tetrahydrocannabinol (THC) Levels and Pharmacodynamic Measures [file jamanetwopen-1-e184841-s002.pdf]

Supplementary Online Content

Spindle TR, Cone EJ, Schlienz NJ, et al. Acute effects of smoked and vaporized cannabis in healthy adults who infrequently use cannabis: a crossover trial. *JAMA Netw Open*. 2018;1(7):e184841. doi:10.1001/jamanetworkopen.2018.4841

eFigure. Mean Whole Blood Δ9-Tetrahydrocannabinol (THC) Concentration Over Time, Across Dose for (A) Smoked and (B) vaporized Conditions
eTable. Correlations (Pearson *r*) Between Individual Change From Baseline Values for Whole Blood Δ9-tetrahydrocannabinol (THC) Levels and Pharmacodynamic Measures

This supplementary material has been provided by the authors to give readers additional information about their work.

© 2018 Spindle TR et al. *JAMA Network Open*.

eFigure. Mean Whole Blood Δ9-Tetrahydrocannabinol (THC) Concentration Over Time, Across Dose for (A) Smoked and (B) Vaporized Conditions

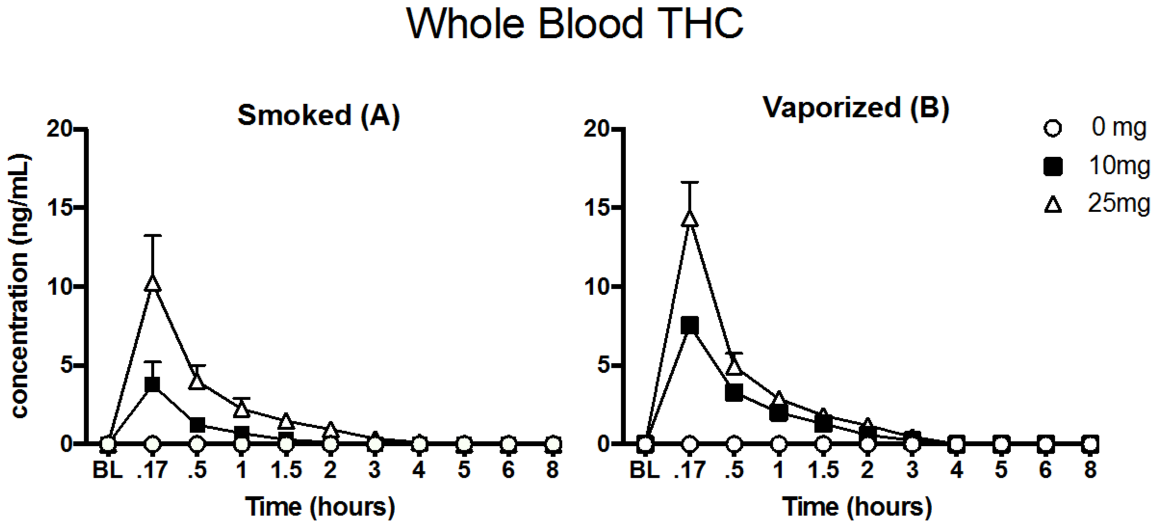

Horizontal axes are not accurate time scales and represent the time points at which blood was sampled. BL=baseline time point; ng/mL=nanograms/milliliter; mg=milligrams.

© 2018 Spindle TR et al. *JAMA Network Open*.

eTable. Correlations (Pearson *r*) Between Individual Change From Baseline Values for Whole Blood Δ9-tetrahydrocannabinol (THC) Levels and Pharmacodynamic Measures

	Whole Blood THC Concentration	
	Smoked	Vaporized
**10mg THC dose**		
Drug Effect (DEQ)	0.38*	0.54*
Heart Rate	0.25*	0.40*
DSST (Total Correct)	-0.06	-0.23
DAT (Distance from stimulus)	0.08	0.05
DAT (Total Correct)	0.07	0.01
PASAT (Total Correct)	0.11	-0.07
**25mg THC dose**		
Drug Effect (DEQ)	0.51*	0.52*
Heart Rate	0.37*	0.37*
DSST (Total Correct)	-0.18	-0.32*
DAT (Distance from stimulus)	0.01	0.14
DAT (Total Correct)	-0.05	-0.14
PASAT (Total Correct)	-0.07	-0.19

Note. Asterisks (*) denote statistical significance (all *p*s<.05). DEQ=Drug Effect Questionnaire; DSST=Digit Symbol Substitution Task; PASAT=Paced Serial Auditory Addition Task"; mg=milligram. A Holm-Bonferroni correction was applied to *p* values for these analyses to reduce the chance of committing a type 1 error.

© 2018 Spindle TR et al. *JAMA Network Open*.
