# Supplementary material for: Acute Effects of Smoked and Vaporized Cannabis in Healthy Adults Who Infrequently Use Cannabis: A Crossover Trial
Source: JAMA Netw Open. 2018 Nov 30;1(7):e184841. doi: 10.1001/jamanetworkopen.2018.4841 (PMC6324384; doi:10.1001/jamanetworkopen.2018.4841)
Supplement: Supplement 3. — Data Sharing Statement [file jamanetwopen-1-e184841-s003.pdf]

Data Sharing Statement

Spindle. Acute Effects of Smoked and Vaporized Cannabis in Healthy Adults Who Infrequently Use Cannabis. *JAMA Netw Open*. Published November 30, 2018. 10.1001/jamanetworkopen.2018.4841

Data
Data available: No
